# Breast Cancer Family History and Behavioral Health Intentions: An Esteem-Relevant Mechanism Informed by the Terror Management Health Model

**DOI:** 10.3390/curroncol32100544

**Published:** 2025-09-28

**Authors:** Emily P. Courtney, Jamie L. Goldenberg

**Affiliations:** 1 Department of Psychology, University of South Florida, Tampa, FL 33620, USA; 2 Department of Health Outcomes and Behavior, Division of Population Science, H. Lee Moffitt Cancer Center and Research Institute, Tampa, FL 33612, USA

**Keywords:** behavioral intentions, breast cancer, cancer, family history, mortality salience, oncology, risk perception, self-esteem, susceptibility, terror management

## Abstract

The terror management health model (TMHM) offers a framework to investigate how concerns about mortality can motivate health-related behaviors through actions that bolster self-esteem. This framework may be especially useful for examining how a family history of breast cancer influences preventative breast health behaviors. Women with no family history, a family history where a family member survived breast cancer, and those who lost a family member to the disease were recruited to participate in one of two preregistered online studies. Participants completed measures of perceived susceptibility, associations of breast cancer with death, breast health esteem, and behavioral breast health intentions. In both studies, the effect of family history on behavioral intentions was serially mediated by susceptibility perceptions, breast cancer–death association, and feelings of esteem related to breast health behaviors. There were no effects of priming mortality. Taken together, the results suggest that both susceptibility perceptions and death associations are critical for encouraging breast health behaviors among women with family history, and this works through a mechanism relevant to self-esteem. Interventions may be more effective when they emphasize the esteem value of breast health behaviors for individuals at increased risk.

## Simple Summary

Despite the importance of prevention and early detection for breast cancer, especially among women who might be at higher risk due to family history, it can still be difficult to prioritize those behaviors in daily life. Family history might also inform how threatening women perceive breast cancer to be. Existing research shows that people are more likely to engage in health behaviors that contribute to their self-esteem. In two studies, we found that women with breast cancer family history perceived their lifetime risk as higher, which predicted how threatening they thought breast cancer was, which predicted breast health self-esteem, which then predicted behavioral intentions. These findings show that women at risk of breast cancer must not only understand their risk but also integrate health behaviors with their self-esteem. These insights can help practitioners communicate that cancer prevention behaviors do contribute to self-esteem, making people more likely to engage in those behaviors.

## 1. Introduction

The average woman has a 1 in 8 chance of being diagnosed with breast cancer at some point in their lifetime, and a 1 in 39 chance of dying from the disease [1]. Breast cancer accounts for 15.5% of new cancer cases in the United States [2]. Further, 15% of women report a family history of breast cancer [3], and familial factors can dramatically increase lifetime risk [4]. Family history increases perceptions of individual susceptibility across a variety of health issues, which influences uptake of preventative health behaviors [5,6,7,8,9]. Individuals, especially those with family history, may also place more value on prevention and detection behaviors [5,6], and especially in the context of breast cancer.

From Breast Cancer Awareness Month to pink ribbons, breast cancer is a disease not only intertwined with personal risk and family history, but with women’s self-esteem [10,11,12]. Generally defined, self-esteem is the combination of mental states which serve to define an individual’s sense of self, for better or worse: the facets of oneself which contribute to one’s idea of their own value [13]. The literature supports positive correlations between self-esteem, body image, and a variety of health indicators: according to Refs. [14,15,16], deriving value from the body and caring for it effectively constitutes a source of self-esteem. Further, positive feelings towards one’s breasts specifically are associated with increased breast self-examination (BSE) behaviors [17]. This research indicates that self-esteem may be *contingent* on caring for the body and its parts (like the breasts). Contingent self-esteem is defined as self-esteem hinged on a self-imposed, personally relevant, and important domain [18]. As such, self-esteem contingent on a healthy body may be especially relevant for individuals with family history of disease, who are then required to put forth more effort to maintain their health. In turn, taking actions oriented toward health may serve as a source of self-esteem among those for whom the actions are critical for staving off disease—especially a disease as publicly visible and socially important as breast cancer. For that reason, the current studies consider breast health esteem as a form of esteem contingency.

Cancer is, of course, associated with death [19,20,21]. Building from a social–psychological existentialist perspective called terror management theory [22], the terror management health model (TMHM [23]) suggests that the uniquely human ability to recognize mortality provokes psychological defenses related to health (i.e., managing terror). The model specifies two routes of defensiveness: when death awareness is conscious, psychological responses aim to remove that awareness (e.g., denial or immediate health actions); conversely, when death thoughts are activated, but not conscious, defensive responses are oriented toward a need for meaning and self-esteem [24,25]. Bolstering feelings of self-esteem thus serves to reduce the “terror” associated with the subconscious awareness of mortality [26]. Prior TMHM-based research has demonstrated that when mortality concerns are activated, behavioral health intentions and behavior [27], including in the domain of breast cancer screening [28], are facilitated through this distal pathway of esteem-related striving. For example, Cooper and colleagues [28] found that variations in death thought accessibility among women at a mammography clinic (a context where death thoughts are likely to be activated [29,30]) interacted with the framing of breast self-exams (BSEs), such that when BSEs were framed as relevant to self-esteem, intentions to conduct BSEs were increased as a function of death awareness. Through this lens and considering potential personal relevance of breast cancer prevention for women with a family history, it follows that heightened awareness of death may increase reliance on breast health behaviors as a means of bolstering esteem, which could promote intentions to engage in those behaviors.

Despite findings that women with a family history perceive themselves as more susceptible and engage in more breast health behaviors, the notion that engagement in those health behaviors could be intertwined with a sense of self-esteem has remained an uninvestigated avenue. Women with a family history of breast cancer may also differ in that they associate the disease with existential threat, which could impact health-related behaviors as contingencies of self-esteem. It follows that the awareness of death should heighten investment in that source of esteem as a means of managing terror among women at risk. The current research examined the extent to which women—especially women with a family history of breast cancer, and especially when that family member died from the disease—perceive themselves as susceptible to breast cancer and associate breast cancer with death.

## 2. Materials and Methods

### 2.1. Purpose and Hypotheses

All data, study materials, and analyses were preregistered on the Open Science Framework (DOI 10.17605/OSF.IO/Z87MF) and all procedures and materials received ethical approval from the Institutional Review Board (STUDY003230; Approved 16 September 2021). All tables and figures are originally produced. Details regarding pilot testing and participant pre-screening can be found in the supplemental materials (Tables S1 and S2).

Study 1 examined perceptions of breast cancer susceptibility, the association between breast cancer and death, breast health esteem contingencies, and breast health intentions as a function family history of breast cancer. We predicted that women with a family history (especially when a family member died from breast cancer) would see themselves as more susceptible to breast cancer (Hypothesis 1) and invest more feelings of esteem in breast health behaviors (Hypothesis 2), which should serially mediate intentions to engage in those behaviors (Hypothesis 3). In addition, we included an exploratory analysis more explicitly investigating death association as a pathway through which breast health esteem could be facilitated. We updated the original preregistration to include this exploratory hypothesis. Other exploratory analyses can be found in the Supplemental Materials (Figures S1–S3; Tables S3–S5). We anticipated that among women with a breast cancer family history, increased susceptibility perceptions would predict increased breast health esteem through increased death associations, which would in turn mediate increased breast health intentions.

Study 2 was designed to more directly investigate the role of death awareness on breast health esteem contingencies and subsequent behavioral intentions with considerations for family history. We hypothesized that, when the awareness of mortality was manipulated, women would report heightened breast health esteem, which would mediate increases in breast health intentions. Family history was expected to moderate this relationship. We also preregistered analyses testing whether the effects of family history on intentions serially mediated by susceptibility perceptions, death association, and breast health esteem, as found in Study 1, would replicate. Additional exploratory analyses are detailed in the Supplementary Materials (Figures S4–S7; Tables S6–S9).

### 2.2. Power Analysis and Participants

For Study 1, power analyses for a small-to-medium effect size for three (family history) groups, and five covariates, and prior research using serial mediation [31], suggested a sample size of 225 participants. A total of 225 cisgender American women aged 40 and participated in exchange for $0.83.

For Study 2, power analyses for a small-to-medium effect size for two experimental groups and a three-group moderator with five covariates [32] suggested a sample size of 450. Responses were collected from 463 participants in exchange for $0.83, but 13 were excluded for failure to pass an attention check, amounting to a final sample of 450 participants.

### 2.3. Measures

In Study 1, participants provided informed consent, being told that they were taking part in a study examining personality and health behavior. All participants completed all measures and attention checks. Materials are described in order of presentation. See Table 1 for all demographics and Table 2 for all descriptive statistics.

Demographics. Participants responded to items pertaining to basic demographics, breast cancer family history status, and past BSE. In line with the preregistration plan, past BSE behaviors, racial/ethnic identity, health insurance status, and education level (SES) were included as covariates in all analyses. In some analyses, prior BSE behaviors served as a significant covariate. No other covariates were significant. Results were unchanged regardless of the BSE covariate’s inclusion across analyses and will not be mentioned further.

Perceived Breast Cancer Susceptibility. Susceptibility was measured with a visual-analog 0–100 scale [33] where participants indicated their subjective lifetime risk of breast cancer, from “0%, no chance of breast cancer” to “100%, definitely will get breast cancer”.

Breast Health Information. Participants were presented with an infographic detailing five behaviors (BSEs, mammograms, clinical breast screenings, and diet and exercise) as important breast cancer risk-reduction measures [34].

Breast Health Esteem. Three items (α= 0.90) adapted from the existing TMHM literature [27,35] measured the degree to which participants derive feelings of esteem from taking care of their breast health (“Taking care of my breast health is an important part of who I am”, “Taking care of my breast health affects how good I feel about myself”, and “Taking care of my breast health allows me to express my competence”.) on 7-point Likert-scale (ranging from ‘strongly disagree’ to ‘strongly agree’). The items were averaged to create a composite breast health esteem score (see Table S10 in the Supplemental Materials for inter-item correlations.)

Breast Health Intentions. Participants were asked to indicate the likelihood that they would engage in the five breast health behaviors listed in the infographic “in the future”, “in the next three months”, and “in the next week” (ranging from 1 = ‘not at all likely’ to 7 = ‘extremely likely’) [36]. The 15-item scale (α= 0.87) was used to create a mean composite breast health intention score (see Table S11 in the Supplemental Materials for items and correlations).

Breast Cancer–Death Association. Participants were asked to rate the extent to which they associated breast cancer with death ranging from 1 (“does not make me think about death at all”) to 7 (“makes me think about death a lot”).

In Study 2, the procedure and materials were the same as in Study 1, with the addition of a mortality salience manipulation [37] aimed at isolating death awareness as a mechanism underlying differences in breast health esteem and intentions. Participants in the mortality salience condition responded to open-ended prompts to “Briefly describe the emotions that the thought of your own death arouses in you” and “Jot down, as specifically as you can, what you think happens to you as you physically die and once you are physically dead”, compared to parallel questions about watching television. This was followed by the same infographic in Study 1 and then the 20-item Positive and Negative Affect Schedule [38], which is commonly used as a delay and distraction task in TMT research [37] to encourage breast health behaviors reaching a distal and thus more self-esteem-contingent status [24].

All the other materials were the same as in Study 1. See Table 1 for demographics and Table 2 for descriptive statistics. 

## 3. Statistics

In Study 1, we investigated three hypotheses. To investigate the first two hypotheses, we conducted a one-way analysis of covariance of the effect of family history group (no family history; family history/survived; family history/died) on the extent to which women perceived themselves as susceptible to breast cancer (Hypothesis 1) and on the extent to which women integrated breast health with feelings of esteem (Hypothesis 2). In Hypothesis 3, we tested a serial mediation model with PROCESS Model 6 in Hayes SPSS 28 plug-in [32]. The serial mediation approach with two mediators began with family history input as the multicategorical predictor variable. In the PROCESS Macro for SPSS 28, multicategorical variables can be classified in different ways (e.g., indicator, sequential, Helmert, etc.) For the purposes of the present studies, and for ease of interpretation, we used the indicator coding scheme for the multicategorical family history predictor variable. The group without any breast cancer family history was thus used as the ‘reference’ or control category, and as such, the other two family history groups were compared to the group without any family history in subsequent comparisons. The groups were coded as follows: no family history = 1, family history/survived = 2, and family history/died = 3. This coding scheme persists throughout all mediation-based analyses. Standardized susceptibility perceptions served as the first mediator, breast health esteem as the second mediator, intentions to engage in breast health behaviors was the dependent outcome variable. For all analyses, indices of effects were estimates with 5000 bootstrap samples, where effects are considered significant if the confidence intervals do not include zero.

**Figure 1:**
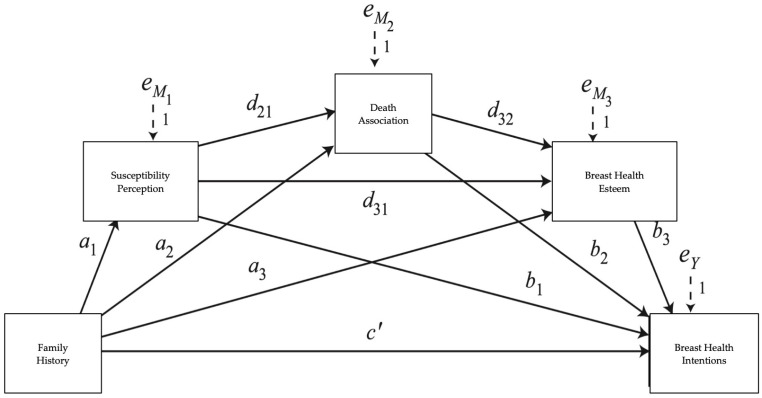
Study 1 exploratory hypothesis PROCESS Model 6 mediation; Study 2 replication analysis.

Finally, in Study 1, the exploratory analysis was also tested with PROCESS Model 6, but with three mediating variables for the indirect effect of family history on breast health intentions. The serial mediation approach with three mediators (perceived susceptibility, followed by death associations, followed by breast health self-esteem) allowed us to examine whether increased susceptibility perceptions, as a function of family history, predicted increased breast health esteem through increased death associations, and this in turn mediated increased breast health intentions. As such, the multicategorical family history group was input as the first independent predictor variable; perceived susceptibility was the first mediating variable, death association was the second mediating variable, breast health esteem was the third mediating variable, and breast health intentions was the dependent outcome variable input in the model. See Figure 1.

In Study 2, we hypothesized that when death was salient, women would report heighted breast health esteem, which would in turn mediate breast health intentions, with family history status serving as a moderator for this relationship. To test this, the mortality salience manipulation was input as the predictor (death versus television, coded as 1 or 0, respectively), breast health esteem as mediator, breast health intentions as the dependent variable, and family history as the multicategorical moderator variable using PROCESS Model 7 for moderated mediation. 

Additionally, in Study 2, we replicated the exploratory model from Study 1: Using PROCESS Model 6 for serial mediation, the multicategorical family history group was input as the first independent predictor variable; perceived susceptibility was the first mediating variable, death association was the second mediating variable, breast health esteem was the third mediating variable, and breast health intentions was the dependent outcome variable input in the model. Again, 5000 bootstrap samples were used to estimate indices of effects, where effects are considered significant if the confidence intervals do not include zero. See Figure 1.

## 4. Results

Study 1, Hypothesis 1. We observed a significant effect of family history status (no family history; family history/survived; family history/died) on the extent to which women perceived themselves as susceptible to breast cancer, *F*(2, 223) = 15.40, *p* < 0.001, $\eta_{p}^{2}\;=0\;.13.$ Both women whose family member died of breast cancer (*N* = 68, *M* = 0.32, *SE* = 0.11) and those whose family member survived breast cancer (*N* = 67, *M* = 0.27, *SE* = 0.11) perceived themselves as more susceptible (both *p*s < 0.001) than those without a family history (*N* = 88, *M* = −0.42, *SE* = 0.10); however, in contrast to the explicit hypothesis involving ordered pairwise susceptibility perceptions, there was no difference between women whose family member died versus survived (*p* = 0.74).

Study 1, Hypothesis 2. In contrast to Hypothesis 2, there was no effect of family history on breast health esteem, *F*(2, 223) = 0.65, *p* = 0.53, $\eta_{p}^{2}=\;0.006.$

Study 1, Hypothesis 3. The relationship between family history and intentions was hypothesized to be mediated by perceptions of susceptibility and breast health esteem. The hypothesized serial mediation was not significant, Hypothesis 3 was not supported, and relevant tables can be found in the online Supplemental Materials.

Study 1, Exploratory Hypothesis (additional exploratory analyses are detailed in the online Supplemental Materials). In an exploratory hypothesis, we examined whether increased susceptibility perceptions, as a function of family history, predicted increased breast health esteem through increased death associations, and this in turn mediated increased breast health intentions. The expected indirect effects for the three-mediator model emerged. The indirect effect of family history through susceptibility, death association, and esteem on breast health intentions was significant for both those with a family history in which a family member survived as well as those with a death in the family compared to those without a family history. See Figure 1 and Table 3.

Study 2, Hypothesis 1: MS effects. We hypothesized that when death was salient, women would report heighted breast health esteem, which would in turn mediate breast health intentions, with family history status serving as a moderator for this relationship. The only significant effect that emerged was an association between breast health esteem and intentions. Thus, the hypothesis was not supported. (Additional analyses pertaining to MS can be found in the online Supplemental Materials). Because none of the hypothesized effects emerged, relevant tables can be found in the Supplemental Materials.

Study 2 Replication Analyses. We performed the additional preregistered analysis to test whether we replicated the mediation findings from Study 1. Susceptibility perceptions, death association, and esteem again mediated the effects of family history on intentions among women whose family member survived as well as among women with a family history in which a family member died. See Figure 1 and Table 4.

## 5. Discussion

In Study 1, women with a family history of breast cancer perceived themselves as more susceptible to the disease (Hypothesis 1), regardless of whether the family member died or survived, but in contrast to Hypothesis 2, there was no direct effect of family history on esteem derived from breast health behavior.

Although the mediation model with only susceptibility perceptions and breast health esteem was not significant and Hypothesis 3 was not supported, the exploratory analyses including breast cancer–death association revealed a meaningful pattern of results. The interplay between susceptibility and death association was critical for all women at heightened breast cancer risk through family history compared to those without family history. Where women with any breast cancer in their family history perceived themselves as susceptible, they also associated breast cancer with death to a greater extent, and in turn were more prone to associate feelings of esteem with taking care of their breast health. Those feelings of esteem then predicted intentions to engage in such behaviors.

In Study 2, we hypothesized that death reminders would be associated with the extent to which breast health conferred self-esteem to facilitate intentions to engage in breast health behaviors. There was no evidence of MS across the board. This could be due to a confounding with family history. The Pilot Study (see online Supplemental Materials) and Study 1 found that women with a family history of breast cancer in which a family member died associated the disease with death to a higher degree. This could suggest that family history alone is a death prime. Combining family history with reminders of death more generally could be conceptual overkill.

Despite the lack of support for the hypothesis concerning mortality salience, the analyses replicated the results of Study 1. Women with a family history of breast cancer (regardless of survival status) saw themselves as more susceptible to breast cancer compared to those without a family history, which related to increased breast cancer–death association, which predicted breast health esteem, which then predicted increased intentions to engage in breast health behavior.

Broadly, these results lend further evidence to the implications of family history for how women view their own risk of breast cancer through an existential lens. Those risk perceptions then influence how important engagement in adaptive breast health behaviors might be to one’s sense of self, which in turn relates to willingness to engage in such behaviors. Although one could interpret the lack of mortality salience effects as evidence against the TMHM, the findings could also be interpreted as follows: women who perceive themselves as more vulnerable (and actually are) on account of their family history respond exactly as the TMHM would predict such that death awareness promotes esteem relevance and corresponding behavior intentions.

Clinical Implications. These studies identified crucial roles of susceptibility perceptions and death associations as facilitators in how women integrated breast behaviors with feelings of esteem. This pattern aligns with health behavior theories, such as the Extended Parallel Process Model [39] and Health Belief Model [40], which emphasize that individuals must feel vulnerable to a health threat and view it as sufficiently serious to motivate protective action. Beyond this, the research highlights the importance of imbuing health behaviors with self-esteem as a key mechanism for fostering behavioral intentions—particularly for women at increased risk due to family history. While many women overestimate their breast cancer risk [41], contributing to overscreening and overdiagnosis [42], these findings suggest that for women at objectively elevated risk potentially due to familial factors, clinicians might more effectively promote preventive behaviors by encouraging women to connect breast health practices with their sense of self-worth.

In continuing to imbue health behaviors with meaning and reconnect breast health with culture, the current studies could inform future breast health communications. Current approaches often downplay the disease’s deadliness [11] while only occasionally framing breast health behaviors as empowering or tied to self-esteem [43]. Additional theorizing suggests that, especially among high-risk women, viewing breast health behaviors as personally important while also psychologically distancing from the existentially troubling physicality of breasts may support healthier risk mitigation behaviors [10]. Ultimately, these studies show that for women at heightened risk, it is pivotal to both connect breast cancer with death and invest personal esteem in health to facilitate adaptive behaviors. These insights could inform a new generation of public health campaigns that highlight both the deadliness of cancer and the esteem gained from caring for one’s health—not only for breast cancer, but for other deadly diseases as well.

Limitations and Strengths. These studies are not without limitations. First, a limitation lies in the breast health intentions variable. Though the reliability for the composite scale was acceptable, intentions may not always map onto actual behavior [44]. Monitoring and measuring actual behaviors instead of behavioral intentions is an important next step [45]. In addition, the measure of breast health esteem is rather broad, despite its use in previous TMHM studies. Going forward, it would also be useful to more finely tune this measure, with additional considerations for self-efficacy and trait-level self-esteem.

Despite preregistration, the sample sizes were informed by the explicit hypotheses, rather than the exploratory analyses, which may be underpowered. Few of the preregistered hypotheses were supported—a conceptual limitation but a strength toward the Open Science movement, especially for social–psychological research [46]. In addition, cross-sectional mediation analyses cannot establish causality [47]. We view our work as an initial step toward identifying mechanisms, noting the replicated mediation model across both studies despite lacking temporal associations. Future studies should employ longitudinal mediation approaches to establish causality and solidify the importance of breast health esteem in managing breast cancer-related terror and facilitating health behaviors.

The present research is bolstered by including women for whom breast cancer is a real threat, particularly those with hereditary risk or family loss. However, it should be expanded to include breast cancer survivors, given the high survival rate for localized breast cancer (99%) [48,49]. Survivors may hold differing health beliefs and perspectives [50] on the deadliness of the disease and fear of recurrence [51,52,53], as well as the extent to which they integrate breast health behaviors, such as continued screening [54], with self-esteem. 

The sample was also predominantly White, highly educated, and insured, likely with adequate access to healthcare services, like clinical breast exams and mammography. This lack of diversity is a major, glaring, and continuing problem in research on hereditary breast cancer, especially for Black women [55,56,57,58], who are diagnosed with more aggressive forms of breast cancer at younger ages [59], and die at higher rates [60]. Encouraging breast health behaviors—potentially through self-esteem—may be especially beneficial for Black women [61,62]. As such, future studies should prioritize groups who could most benefit from improved breast health behavior uptake. 

## 6. Conclusions

Despite medical advances, breast cancer is not a disease that will likely ever be eradicated, or even fully curable or preventable; some people are at significantly higher risk than others. These studies provide support for an existential position that, for those at higher risk, mortality concerns tied to breast cancer and self-esteem linked to breast health behaviors are important factors for promoting risk-reduction and early-detection behaviors that improve prognoses and survivorship.

## Figures and Tables

**Table 1:** Study demographics.

Item	Study 1 *n*	Study 1%	Study 2 *n*	Study 2%
Racial/Ethnic Identity				
Asian American/Pacific Islander	4	1.8	8	1.8
Native American/Alaska Native	1	0.4	3	0.7
Black/African American	13	5.8	23	5.1
White	195	86.7	393	87.3
Hispanic/Latinx	5	2.2	11	2.4
Multiple/Biracial	6	2.7	11	2.4
Self-Identify	1	0.4	1	0.2
Sexual Orientation				
Straight	206	91.6	396	88.0
Lesbian	2	0.9	1	0.2
Bisexual	10	4.4	11	2.4
Asexual	3	1.3	28	6.2
Pansexual	1	0.4	5	1.1
Self-Identify	3	1.3	6	1.0
Health Insurance				
Yes	198	88.0	408	90.9
No	24	10.7	41	9.1
Not Sure	3	1.3	1	0.2
Education Level				
Some high school	1	0.4	2	0.4
High school diploma/GED	53	23.6	115	25.6
Associate’s degree	29	12.9	66	14.7
Bachelor’s degree	82	36.4	160	35.6
Master’s degree	36	16.0	73	16.2
Technical degree	6	2.7	9	2.0
Professional degree	11	4.9	18	4.0
Doctorate	7	3.1	7	1.6
Family History Status				
No family history	90	40.0	183	40.7
Yes, and those diagnosed with breast cancer survived	67	29.8	140	31.1
Yes, and at least one person diagnosed with breast cancer passed away as a result of the disease	68	30.2	127	28.2
Relationship to Person with Breast Cancer (only presented to those who indicated family history to previous item, *N* = 135, could select all that apply)				
First-degree family member	54		110	
Second-degree family member	94		174	
Further removed (e.g., great grandparent)	13		26	
Time since death of family member (only presented to those who indicated that a family member diagnosed with breast cancer died as a result of the disease, *N* = 68)				
Less than 1 year	1	1.5	5	3.9
1–5 years	9	13.2	21	16.5
5–10 years	11	16.2	26	20.5
10+ years	47	69.1	75	59.1
Genetic Testing for Breast Cancer Risk				
No	203	90.2	404	89.8
Yes, but tested negative	19	8.4	41	9.1
Yes, and tested positive for at least one genetic risk factor	3	1.3	5	1.1

**Table 2:** Descriptive Statistics.

Descriptive Statistics	Study 1			Study 2		
	M (*SD*)	Skewness (*SE*)	Kurtosis (*SE*)	M (*SD*)	Skewness (*SE*)	Kurtosis (*SE*)
Age	52.38 (*9.26*)	0.66 (*0.16*)	−0.18 (*0.32*)	52.66 (*9.17*)	0.47 (*0.11*)	−0.62 (*0.23*)
Past BSE Behaviors	2.39 (*1.35*)	1.29 (*0.16*)	0.73 (*0.32*)	2.43 (*1.36*)	1.22 (*0.11*)	0.69 (*0.23*)
Susceptibility	39.04 (*22.21*)	0.12 (*0.16*)	−0.83 (*0.32*)	40.57 (*24.41*)	0.22 (*0.12*)	−0.70 (*0.23*)
Breast Health Esteem	4.49 (*1.54*)	−0.13 (*0.16*)	−0.71 (*0.32*)	4.30 (*1.65*)	−0.07 (*0.12*)	−0.77 (*0.23*)
Breast Health Intentions	4.73 (*1.13*)	−0.07 (*0.16*)	−0.38 (*0.32*)	4.50 (*1.08*)	−0.28 (*0.12*)	−0.12 (*0.23*)
Breast Cancer–Death Association	4.47 (*1.82*)	−0.35 (*0.16*)	−0.81 (*0.32*)	4.34 (*1.88*)	−0.23 (*0.12*)	−0.99 (*0.23*)

**Table 3:** Study 1 exploratory hypothesis PROCESS Model 6 mediation. Controlling for age, race, insurance status, SES, and past BSE behaviors, N = 223. Estimates of indirect effects for serial mediation model.

PROCESS Model 6, Study 1				Effect	SE	LLCI	ULCI
	Indirect Effects						
		Family history → Susceptibility → Intentions (*a*_1_*b*_1_)					
			No family history vs. Survived	−0.003	0.055	−0.101	0.115
			No family history vs. Died	−0.003	0.058	−0.113	0.121
		Family history → Death → Intentions (*a*_2_*b*_2_)					
			No family history vs. Survived	−0.015	0.024	−0.067	0.030
			No family history vs. Died	0.040	0.035	−0.010	0.120
		Family history → Esteem → Intentions (*a*_3_*b*_3_)					
			No family history vs. Survived	0.022	0.060	−0.080	0.157
			No family history vs. Died	−0.006	0.053	−0.113	0.100
		Family history → Susceptibility → Death → Intentions (*a*_1_*d*_21_*b*_2_)					
			No family history vs. Survived	0.019	0.015	−0.006	0.053
			No family history vs. Died	0.021	0.016	−0.006	0.057
		Family history → Susceptibility → Esteem → Intentions (*a*_1_*d*_31_*b*_3_)					
			No family history vs. Survived	0.016	0.019	−0.020	0.056
			No family history vs. Died	0.017	0.020	−0.021	0.059
		Family history → Death → Esteem → Intentions (*a*_2_*d*_32_*b*_3_)					
			No family history vs. Survived	−0.011	0.017	−0.051	0.015
			**No family history vs. Died**	**0.029**	**0.017**	**0.001**	**0.068**
		Family history → Susceptibility → Death → Esteem → Intentions (*a*_1_*d*_21_*d*_32_*b*_3_)					
			**No family history vs. Survived**	**0.014**	**0.008**	**0.003**	**0.035**
			**No family history vs. Died**	**0.015**	**0.009**	**0.003**	**0.037**

*Note:* 95% confidence intervals (CI) that do not include zero are considered statistically significant and denoted in **bold**. For comparisons of family history, no family history is coded as 1, family history: survived is coded as 2, and family history: died is coded as 3. The no family history group is treated as the reference category in this analysis, so the other groups are compared to that reference category. Estimates of direct effects can be found in online Supplemental Materials. LLCI: Lower Limit Confidence Interval; ULCI: Upper Limit Confidence Interval; SE = standard error.

**Table 4:** Study 2 replication analysis PROCESS model 6 mediation. Controlling for age, race, insurance status, SES, and past BSE behaviors, N = 447. Estimates of indirect effects for serial mediation model.

PROCESS Model 6, Study 2 Replication Analysis				Effect	SE	LLCI	ULCI
	Indirect Effects						
		Family history → Susceptibility → Intentions (*a*_1_*b*_1_)					
			No family history vs. Survived	−0.057	0.029	−0.118	−0.005
			**No family history vs. Died**	**−0.080**	**0.040**	**−0.162**	**−0.007**
		Family history → Death → Intentions (*a*_2_*b*_2_)					
			No family history vs. Survived	−0.007	0.010	−0.032	0.009
			No family history vs. Died	0.012	0.013	−0.008	0.043
		Family history → Esteem → Intentions (*a*_3_*b*_3_)					
			No family history vs. Survived	0.006	0.049	−0.090	0.102
			No family history vs. Died	−0.023	0.057	−0.134	0.091
		Family history → Susceptibility → Death → Intentions (*a*_1_*d*_21_*b*_2_)					
			No family history vs. Survived	0.011	0.009	−0.007	0.030
			No family history vs. Died	0.016	0.013	−0.010	0.042
		Family history → Susceptibility → Esteem → Intentions (*a*_1_*d*_31_*b*_3_)					
			No family history vs. Survived	0.010	0.014	−0.015	0.038
			No family history vs. Died	0.014	0.019	−0.022	0.053
		Family history → Death → Esteem → Intentions (*a*_2_*d*_32_*b*_3_)					
			No family history vs. Survived	−0.012	0.012	−0.036	0.011
			No family history vs. Died	0.020	0.013	−0.003	0.050
		Family history → Susceptibility → Death → Esteem → Intentions (*a*_1_*d*_21_*d*_32_*b*_3_)					
			**No family history vs. Survived**	**0.018**	**0.007**	**0.008**	**0.034**
			**No family history vs. Died**	**0.026**	**0.009**	**0.011**	**0.047**

*Note:* 95% confidence intervals (CI) that do not include zero are considered statistically significant and denoted in **bold**. For comparisons of family history, no family history is coded as 1, family history: survived is coded as 2, and family history: died is coded as 3. The no family history group is treated as the reference category in this analysis, so the other groups are compared to that reference category. Estimates of direct effects can be found in online Supplemental Materials. LLCI: Lower Limit Confidence Interval; ULCI: Upper Limit Confidence Interval; SE = standard error.

## Data Availability

Preregistration information and the data that support the findings of this study are openly available on the Open Science Framework at [https://osf.io/z87mf/?view_only=f882bcac4c2c468fa9413bbcd28d7751], reference number [DOI 10.17605/OSF.IO/Z87MF].

## Supplementary Materials

The following supporting information can be downloaded at: https://www.mdpi.com/article/10.3390/curroncol32100544/s1, Supplemental citations listed [1,3,19,35,36,63,64,65,66,67,68,69,70,71]. Table S1. Pilot Study Demographics (N = 626). Table S2. Pilot Study Descriptive Statistics. Table S3: Study 1 Hypothesis 3 PROCESS Model 6 Mediation. Table S4. Study 1 Exploratory PROCESS Model 6 (Death Association in place of Susceptibility) Estimates of effects for serial mediation model. Table S5. Study 1 Exploratory PROCESS Model 6 (Death First). Table S6: Study 2 PROCESS Model 7: Moderated Mediation: MS effects. Table S7. Study 2 PROCESS Model 83 (Death First). Table S8. Study 2 PROCESS Model 83 (Susceptibility First). Table S9. Study 2 Replication, PROCESS Model 6 (Death First). Table S10: Breast Health Esteem Correlations. Table S11: Breast Health Intentions Correlations. Figure S1: Study 1 Hypothesis 3, PROCESS Model 6. Figure S2. Study 1 Exploratory PROCESS Model 6 (Death Association in place of Susceptibility). Figure S3. Exploratory PROCESS Model 6 (Death First). Figure S4: Study 2 PROCESS Model 7: Moderated Mediation: MS Effects. Figure S5. Study 2 PROCESS Model 83 (Death First). Figure S6. Study 2 PROCESS Model 83 (Susceptibility First). Figure S7. Study 2 Replication, PROCESS Model 6 (Death First).
