# Supplementary material for: Breast Cancer Family History and Behavioral Health Intentions: An Esteem-Relevant Mechanism Informed by the Terror Management Health Model
Source: Curr Oncol. 2025 Sep 28;32(10):544. doi: 10.3390/curroncol32100544 (PMC12563849; doi:10.3390/curroncol32100544)
Supplement: Supplementary file 1 [file curroncol-32-00544-s001.zip › curroncol-3817071-supplementary.pdf]

Supplemental Materials

Pilot Testing

All pilot testing, prescreening, and subsequent study materials were approved by the institutional review board (STUDY3230). Additionally, all data, study materials, and analyses were preregistered on the Open Science Framework (https://osf.io/z87mf/?view_only=f882bcac4c2c468fa9413bbcd28d7751, BLINDED) and are freely available.

A first step to this series of studies was to determine the extent to which family history plays a role in the perception of existential threat associated with health problems. That is, we wanted to first establish whether family history influences the extent to which health problems, including breast cancer, are associated with death. Data were collected from 626 undergraduate students (M_{age} = 20.59, SD_{age} = 3.90; see Table S1 for demographics) who were first presented with an informed consent and then asked to rate the association of health problems with death 5-point Likert scale ranging from 1 ("*does not make me think about death at all*") to 5 ("*makes me think about death a lot*")[1].

[1] In this pilot study, 24 health issues were assessed, including but not limited to breast cancer, testicular cancer, lung cancer, skin cancer, asthma, and dental problems (the last two of which have been used as control conditions in other terror management literature(Arndt et al., 2003, 2007). However, breast cancer is the health issue of interest and as such, the other health problems will not be mentioned further.

Table S1. *Pilot Study Demographics (N=626)*

Item	*n*	%
Gender		
Man	154	22.80
Woman	458	68.70
Transman	2	0.10
Transwoman	1	0.10
Non-Binary	7	1.00
Self-Identify	4	6.00
Race/Ethnicity		
Asian American/Pacific Islander	61	9.0
Native American/Alaska Native	3	0.40
White	276	40.80
Black/African American	68	10.10
Hispanic/Latinx	140	20.70
Arab/Middle Eastern	17	2.50
Biracial	31	4.60
Self-Identify	30	4.80

Across all reported genders, these data presented an ordered pattern of results in terms of the extent that breast cancer was associated with death based on family history, $F(2, 623) = 9.85$, $p < 0.001$, $\eta_p^2 = 0.031$. LSD pairwise comparisons indicated that breast cancer-death associations did not differ between individuals without a family history ($N = 411$, $M = 3.77$, $SD = 1.18$) and individuals with a family history in which a family member survived ($N = 133$, $M = 3.90$, $SD = 1.01$; $p = 0.24$). However, those with a family history in which a family member died as a result of breast cancer ($N = 82$, $M = 4.37$, $SD = 0.85$) associated breast cancer with death to a greater extent than both those without a family history ($p < 0.001$) and those with a family history with survival ($p = 0.003$).

Analyzing only women, we observed the same pattern of results, $F(2, 453) = 7.71$, $p < 0.001$, $\eta_p^2 = 0.033$. Breast cancer-death associations did not differ for those without a family history ($N = 283$, $M = 3.90$, *SD*

= 1.12) and those whose family member survived ((*N* = 107, *M* =4.02, *SD* = 0.96; $p = 0.30$). Women with a family history in which a family member died as a result of breast cancer (*N* = 66, *M* =4.45, *SD* = 0.75) associated breast cancer with death to a greater extent than both those without a family history ($p < 0.001$) and those whose family member survived ($p = 0.008$). See Table S2 for sample sizes, means, and standard deviations.

Table S2. *Pilot Study Descriptive Statistics*

Table S2

Pilot Study Descriptive Statistics for Association Between Breast Cancer and Death

	All Participants			Only Women		
	M	*SD*	*N*	*M*	*SD*	*N*
No Family History	3.77	1.18	411	3.90	1.12	283
Family History: Survived	3.90	1.01	133	4.02	0.96	107
Family History: Died	4.37**	0.85	82	4.45*	0.75	66

Note: * Indicates $p < 0.05$; ** indicates $p < 0.001$

These findings align with studies from Padamasee and colleagues (Padamsee et al., 2020), where women with a close family history involving death perceived breast cancer as more traumatic, which may imply that family history can influence perceptions of existential threat associated with the disease. This information was used to guide mortality salience priming materials in the present research. First, in Study 1, having had a family member die of breast cancer implies increased death salience, which should impact perceptions of susceptibility and the extent to which breast cancer would be associated with death. As such, we used the same question from pilot testing in which women were asked about how much breast cancer made them think about death in both studies. Additionally, in Study 2, given that breast cancer is associated with death to a differential degree depending on family history, rather than prime breast cancer alone as an existential threat (Morris et al., 2013), death itself was made explicit so as to make existential threat uniform across family history groups, with family history expected to serve as a moderating variable.

These data were used to inform the way family history-related questions were approached in the current study. All family history questions asked individuals to state whether they had a family history of breast cancer,

and whether they had a death in the family caused by breast cancer. Further, 34.34% of respondents reported a family history of breast cancer, and 13.10% reported the death of a family member due to breast cancer. These data were synthesized with information from Brewer and colleagues (Brewer et al., 2017) and Ramsey and colleagues (Ramsey et al., 2006) to inform prescreen sample sizes. We oversampled in prescreening by a degree of at least 20% to attain a reasonable number of participants with both a family history and with a family history where a family member died of breast cancer.

Prescreening

A shortcoming in existing TMHM literature concerns the samples that have been used for research on breast cancer. Undergraduate student samples have often been studied in prior research, but the use of convenience samples is problematic due to the fact that younger women are simply not as vulnerable to breast cancer, and risk increases with age (Howlader et al., 2019). Additionally, doctors typically recommend that women of average risk level begin mammogram screenings at age 40 (*American Cancer Society*, n.d.). As such, women over age 40 were the specific target group for the purposes of the present studies. Integrating the data from the pilot studies, established work on hereditary breast cancer incidence, and reported rates of breast cancer family history/deaths in family, we recruited 2500 participants via the Prolific online survey platform to attain a sufficiently large pool of potential participants.

The one-minute prescreen posting was available to Prolific workers who indicated in preliminary screening through the survey platform that they 1) lived in the United States; 2) were assigned female at birth; 3) indicated cisgender woman as their gender identity; and 4) were between 40 and 100 years of age. Participants were paid $.016 for their participation in the prescreen survey.

In the prescreen survey, participants were presented with an informed consent. Then, participants were asked whether they had a family history of breast cancer using the same question from pilot testing (i.e., “Do you have a family history of breast cancer,” with responses “No,” “Yes, and those diagnosed with breast cancer survived,” and “Yes, and at least one person diagnosed with breast cancer passed away as a result of the

disease.")[2]. Lastly, participants were asked if they had ever been diagnosed with breast cancer themselves. Prolific Worker ID numbers were also collected through the Prolific platform for the purposes of inviting specific workers back to participate in the main studies. Worker ID numbers and locations have been redacted from open data for the sake of participant anonymity and confidentiality.

Including incomplete data, a total of 2519 participants responded to the Prolific posting. After removing incomplete responses, the total sample for inclusion consideration in both Studies 1 and 2 amounted to 2497. Each participant was assigned a unique identifying number. Then, those who reported having had breast cancer themselves (n = 82) were excluded for a sample of 2415 cisgender American women between 40 and 100 without any personal history of breast cancer. We then assigned a new unique identifier to each participant and divided them into three groups based on family history (no family history, n = 1657; family history/survived, n = 435; family history/dead, n = 323). From there, each participant within each group was assigned another unique identifying number for the group, ranging from one up through the total number of participants in that given family history group.

In order to balance the family groups in the main studies, a random number generator (using the "random" package in R) was used to select participants with corresponding ID numbers for inclusion. Participants could be randomly selected to participate in either Study 1 or Study 2, but not both. Three random number generation RMarkdown documents (see Appendix A) reflect the R code used, as well as the random numbers output by the code via RMarkdown. The "random" package had a shortcoming in that it did output random numbers with replacement, lending to the potential for duplicates. In the event of duplicates, the next available ID number either immediately before (heads) or immediately after (tails) the given number was selected instead based on a coin flip. Based on that random selection, individual participants were recruited for participation for either Study 1 or Study 2 via the Prolific platform using their Prolific Worker IDs.

Study 1, Hypothesis 3 Supplemental Figure and Table

Figure S1: Study 1 Hypothesis 3, PROCESS Model 6

[2] Also in this section, participants who responded that they had some sort of family history were asked to indicate their relationship to the person/people diagnosed with breast cancer (i.e., first-degree, second-degree, or further-removed family member). Participants who indicated that a family member had passed away from breast cancer were also asked to indicate how long it had been since their most recent family member passed away. These items were preregistered, but sample sizes were too small to make meaningful conclusions.

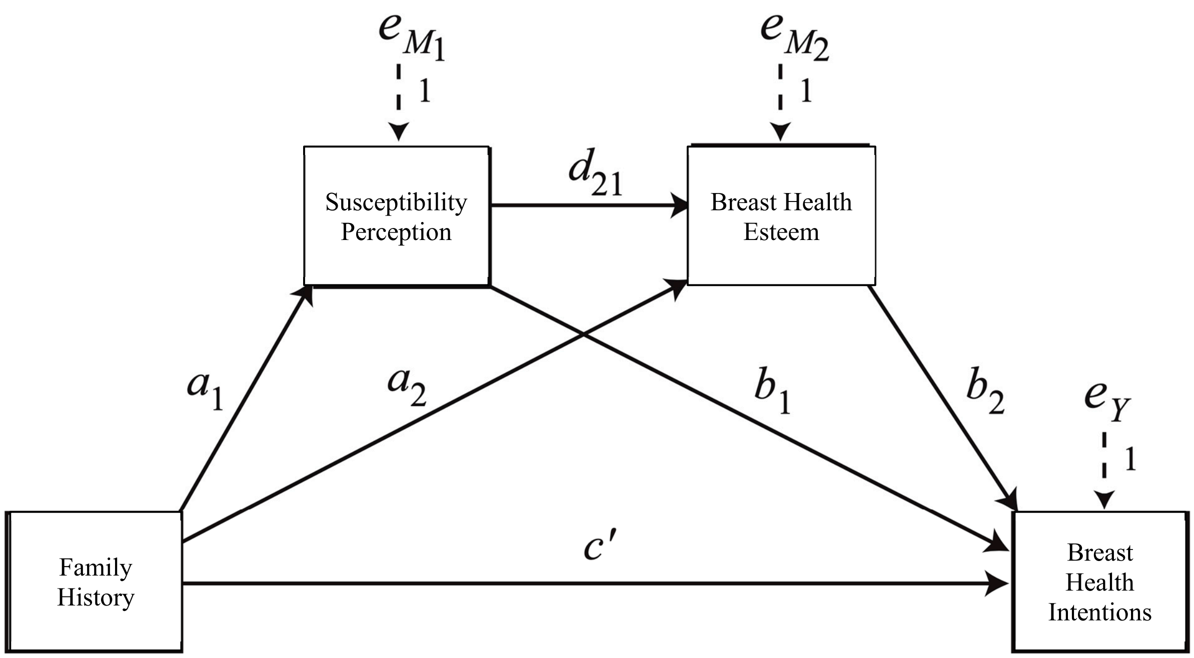

Table S3: Study 1 Hypothesis 3 PROCESS Model 6 Mediation

Estimates of effects for serial mediation model
Controlling for age, race, insurance status, SES, and past BSE behaviors, N = 223

PROCESS Model 6	effect	SE	LLCI	ULCI
Direct Effects on Susceptibility				
Family History (a_1)				
**No family history vs. Survived**	**0.690**	**0.153**	**0.389**	**0.991**
**No family history vs. Died**	**0.743**	**0.152**	**0.444**	**1.043**
Direct Effects on Breast Health Esteem				
Family History (a_2)				
No family history vs. Survived	0.051	0.249	−0.441	0.542
No family history vs. Died	0.105	0.250	−0.388	0.598
Susceptibility (d_{21})	0.205	0.106	−0.005	0.414
Direct Effects on Intentions				
Family History (c')				
No family history vs. Survived	00.202	0.169	−0.132	0.536
No family history vs. Died	0.107	0.170	−0.228	0.442
Susceptibility (b_1)	0.020	0.073	−0.124	0.164
**Breast Health Esteem (b_2)**	**0.233**	**0.047**	**0.142**	**0.325**
Indirect Effects				
Family history → Susceptibility → Intentions (a_1b_1)				
No family history vs. Survived	0.033	0.020	−0.002	0.078
No family history vs. Died	0.036	0.021	−0.003	0.083
Family history → Esteem → Intentions (a_2b_2)				
No family history vs. Survived	0.012	0.065	−0.102	0.150
No family history vs. Died	0.025	0.062	−0.095	0.150
Family history → Susceptibility → Esteem → Intentions ($a_1d_{21}b_2$)				
No family history vs. Survived	0.033	0.020	−0.002	0.078
No family history vs. Died	0.036	0.021	−0.003	0.083

Note: 95% confidence intervals (CI) that do not include zero are considered statistically significant and denoted in **bold**. For comparisons of family history, no family history is coded as 1, family history: survived is coded as 2, and family history: died is coded as 3. The no family history group is treated as the reference category in this analysis, so the other groups are compared to that reference category.

Additional Study 1 Exploratory Analyses

In the preregistration plan, we detailed that exploratory analyses would incorporate associations between death and breast cancer. First, we aimed to replicate the Pilot Study and determine if women with a family history of breast cancer differed in the extent to which they associated the disease with death, especially women who lost a family member. Additionally, we investigated death association in place of susceptibility perceptions as a mediator, and also employed both death association and susceptibility perception variables as serial mediators on the effects of family history on breast health esteem, and in turn, breast health intentions when controlling for age, race, insurance status, SES, and prior BSE behaviors.

To investigate whether the Pilot Study replicated, we employed the same one-way between-subject ANCOVA to analyze effects of family history on associations between death and breast cancer while continuing to control for SES, past BSE behaviors, insurance status, race, and age. Again, a significant main effect of family history ($F[2, 223] = 6.41$, $p = .002$, $\eta_p^2 = 0.06$), and the anticipated pattern of LSD pairwise comparisons as informed by pilot data, emerged. Breast cancer-death associations did not differ between women without a family history (*N* = 88, *M* = 4.22, *SD* = 1.96) and those whose family member survived ((*N* = 67, *M* = 4.23, *SD* = 1.69; *p* = 0.83), whereas women whose family member died (*N* = 68, *M* = 5.12, *SD* = 1.59) associated breast cancer with death significantly more than both those whose family member survived (*p* = 0.004) and those without a family history (*p* = 0.001).

Then, we investigated the role of death association in place of the susceptibility perception variable in a PROCESS Model 6 for serial mediation, parallel to the analysis used to test Hypothesis 3. Family history was still used as the predictor, death associations replaced susceptibility perceptions to serve as the first mediator, breast health esteem as the second mediator, and breast health intentions as the outcome variable. First, there was a significant direct effect of family history on death associations, but only among those with a family history in which a person died, *B* = 0.94, *SE* = 0.29, 95%CI [.38, 1.51]. This finding reflects the ANCOVA, wherein those whose family member died of breast cancer associated the disease with death to a greater extent than the other groups, who did not differ from each other. Further, there was a significant direct effect of death association on breast health esteem, *B* = 0.23, *SE* = 0.05, 95%CI [.12, .34] and a direct effect of breast health

esteem on breast health intentions, *B* = 0.21, *SE* = 0.05, 95%CI [0.12, 31]. In addition, there was a significant serial indirect effect of family history on intentions through death associations and esteem among those with a family history in which someone died, *B* = 0.05, *SE* = 0.02, 95%CI [0.01, 0.10]. This indirect effect suggests that, among women whose family member died of breast cancer, associating the disease with death and placing feelings of esteem in breast health behaviors to a greater extent mediates intentions to engage in breast health behaviors.

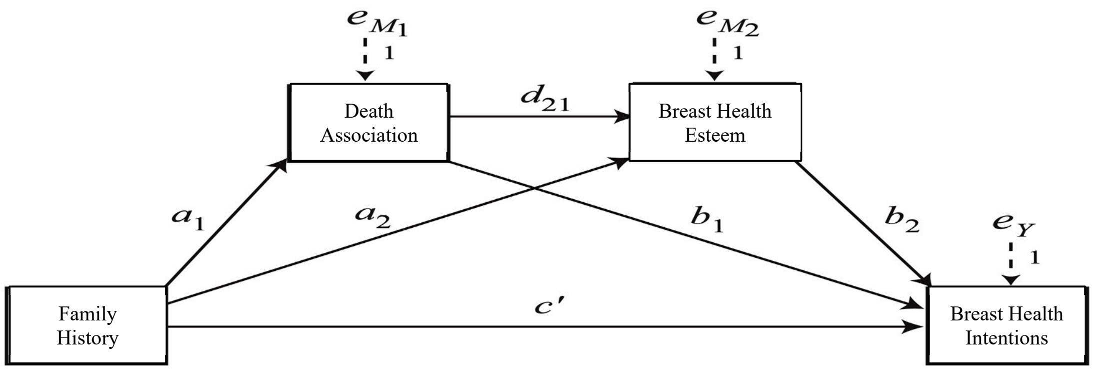

Figure S2. *Study 1 Exploratory PROCESS Model 6 (Death Association in place of Susceptibility)*

Table S4. *Study 1 Exploratory PROCESS Model 6 (Death Association in place of Susceptibility)*
Estimates of effects for serial mediation model
Controlling for age, race, insurance status, SES, and past BSE behaviors, N = 223

PROCESS Model 6	effect	SE	LLCI	ULCI
Direct Effects on Death Association				
Family History (a_1)				
No family history vs. Survived	0.063	0.288	−0.505	0.630
**No family history vs. Died**	**0.946**	**0.287**	**0.381**	**1.511**
Direct Effects on Breast Health Esteem				
Family History (a_2)				
No family history vs. Survived	0.177	0.231	−0.278	0.632
No family history vs. Died	0.039	0.236	−0.425	0.503
**Death Association (d_{21})**	**0.231**	**0.055**	**0.123**	**0.339**
Direct Effects on Intentions				
Family History (c')				
No family history vs. Survived	0.216	0.161	−0.102	0.533
No family history vs. Died	0.068	0.164	−0.256	0.391
Death Association (b_1)	0.063	0.040	−0.016	0.141
**Breast Health Esteem (b_2)**	**0.214**	**0.048**	**0.120**	**0.308**
Indirect Effects				
Family history → Death Association → Intentions (a_1b_1)				
No family history vs. Survived	0.004	0.022	−0.035	0.058
No family history vs. Died	0.059	0.045	−0.016	0.159
Family history → Esteem → Intentions (a_2b_2)				
No family history vs. Survived	0.038	0.067	−0.059	0.169
No family history vs. Died	0.008	0.050	−0.088	0.114
Family history → Death Association → Esteem → Intentions ($a_1d_{21}b_2$)				

No family history vs. Survived	0.003	0.015	−0.029	0.034
**No family history vs. Died**	**0.047**	**0.021**	**0.013**	**0.094**

Note: 95% confidence intervals (CI) that do not include zero are considered statistically significant and denoted in **bold**. For comparisons of family history, no family history is coded as 1, family history: survived is coded as 2, and family history: died is coded as 3. The no family history group is treated as the reference category in this analysis, so the other groups are compared to that reference category.

In the updated preregistration, we also detailed two three-variable serial mediation models employing both the susceptibility perception and death association variables. Two additional PROCESS Model 6 analyses for serial mediation with three mediators were conducted: one with death association as the first mediator and susceptibility perception as the second, and one with susceptibility perception as the first mediator and death association as the second. In both analyses, breast health esteem served as the third mediator and breast health intentions as the outcome variable of interest, controlling for age, race, insurance status, SES, and prior BSE behaviors. The susceptibility-first model is reported in the main manuscript (see Study 1 Exploratory Hypothesis), and the death-association-first model is reported here.

In this model, death association was input as the first mediator, susceptibility perceptions as the second, and esteem as the third. A few direct effects emerged. There was a significant direct effect of family history on death association only among women whose family member died, $B = 0.95$, $SE = 0.29$, 95%CI [0.38, 1.51]. There were also direct effects of family history on susceptibility perception among women whose family member survived ($B = 0.68$, $SE = 0.15$, 95%CI [0.39, 0.98] and women whose family member died ($B = 0.63$, $SE = 0.15$, 95%CI [0.33, 0.93]). Further, there was a direct effect of death association on susceptibility perception, $B = 0.13$, $SE = 0.04$, 95%CI [0.06, 0.19]. On the breast health esteem variable, there was a direct effect of death association, $B = 0.22$, $SE = 0.06$, 95%CI [0.11, 0.33]. Lastly, there was a direct effect of breast health esteem on breast health intentions, $B = 0.21$, $SE = 0.05$, 95%CI [0.12, 0.31]

In line with the prior analysis in which death association and esteem were the only two mediators in the model, the only significant indirect effect was a basic replication of that prior analysis. Among those with a family history in which someone died compared to those without a family history, there was a significant indirect effect of family history on intentions through death associations and breast health esteem, $B = 0.02$, *SE*

= 0.01, 95%CI [0.01, 0.09]. The same effect did not manifest in comparing those whose family member survived to those without a family history.

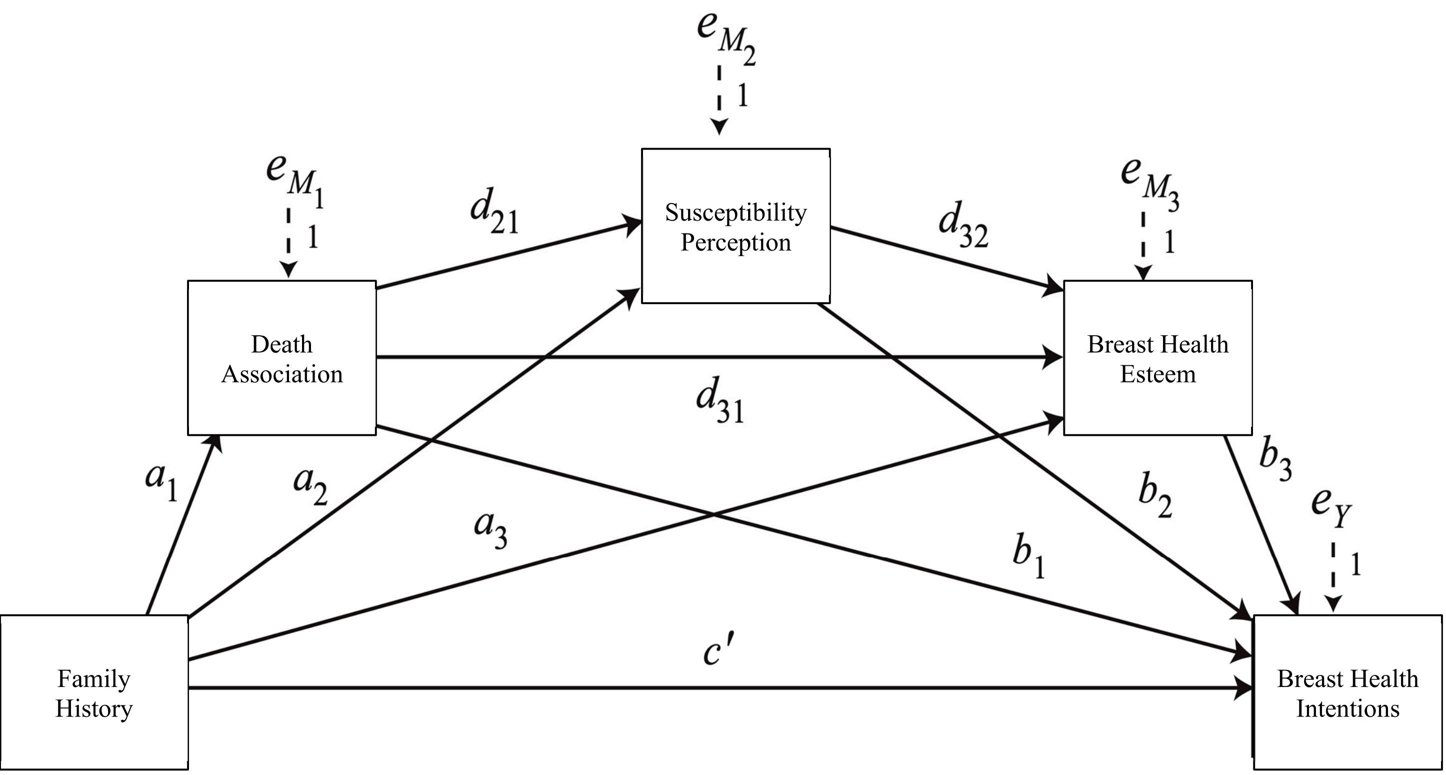

Figure S3. *Exploratory PROCESS Model 6 (Death First)*

Table S5. *Study 1 Exploratory PROCESS Model 6 (Death First)*

Controlling for age, race, insurance status, SES, and past BSE behaviors, N = 223
Estimates of effects for serial mediation model.

PROCESS Model 6	effect	SE	LLCI	ULCI
Direct Effects on Death Association				
Family History (a_1)				
No family history vs. Survived	0.063	0.288	−0.505	0.630
**No family history vs. Died**	**0.946**	**0.287**	**0.381**	**1.512**
Direct Effects on Susceptibility Perceptions				
Family History (a_2)				
**No family history vs. Survived**	**0.682**	**0.149**	**0.389**	**0.976**
**No family history vs. Died**	**0.626**	**0.152**	**0.326**	**0.925**
**Death Association (d_{21})**	**0.125**	**0.035**	**0.055**	**0.194**
Direct Effects on Breast Health Esteem				
Family History (a_3)				
No family history vs. Survived	0.103	0.242	−0.373	0.580
No family history vs. Died	−0.029	0.245	−0.511	0.453
**Death Association (d_{31})**	**0.217**	**0.056**	**0.106**	**0.328**
Susceptibility Perceptions (d_{32})	0.108	0.106	−0.101	0.317
Direct Effects on Breast Health Intentions				
Family History (c')				
No family history vs. Survived	0.218	0.169	−0.115	0.552

No family history vs. Died	0.070	0.171	−0.267	0.407
Death Association (b_1)	0.063	0.041	−0.017	0.143
Susceptibility Perceptions (b_2)	−0.004	0.074	−0.151	0.143
**Breast Health Esteem (b_3)**	**0.214**	**0.048**	**0.120**	**0.309**
Indirect Effects				
Family history → Death Association → Intentions (a_1b_1)				
No family history vs. Survived	0.004	0.022	−0.034	0.059
No family history vs. Died	0.060	0.046	−0.016	0.163
Family history → Susceptibility → Intentions (a_2b_2)				
No family history vs. Survived	−0.003	0.056	−0.110	0.120
No family history vs. Died	−0.003	0.051	−0.103	0.107
Family history → Esteem → Intentions (a_3b_3)				
No family history vs. Survived	0.022	0.060	−0.084	0.154
No family history vs. Died	−0.006	0.056	−0.117	0.109
Family history → Death → Susceptibility → Intentions ($a_1d_{21}b_2$)				
No family history vs. Survived	0.000	0.003	−0.007	0.006
No family history vs. Died	−0.001	0.010	−0.022	0.021
Family history → Death → Esteem → Intentions ($a_1d_{31}b_3$)				
No family history vs. Survived	0.003	0.014	−0.027	0.032
**No family history vs. Died**	**0.044**	**0.021**	**0.012**	**0.092**
Family history → Susceptibility → Esteem → Intentions ($a_2d_{32}b_3$)				
No family history vs. Survived	0.016	0.018	−0.020	0.055
No family history vs. Died	0.015	0.017	−0.018	0.051
Family history → Death Association → Susceptibility → Esteem → Intentions ($a_1d_{21}d_{32}b_3$)				
No family history vs. Survived	0.000	0.001	−0.002	0.003
No family history vs. Died	0.003	0.003	−0.003	0.010

Note: 95% confidence intervals (CI) that do not include zero are considered statistically significant and denoted in **bold**. For comparisons of family history, no family history is coded as 1, family history: survived is coded as 2, and family history: died is coded as 3. The no family history group is treated as the reference category in this analysis, so the other groups are compared to that reference category.

Study 2 MS Effects Tables and Figures

Figure S4: Study 2 PROCESS Model 7: Moderated Mediation: MS Effects

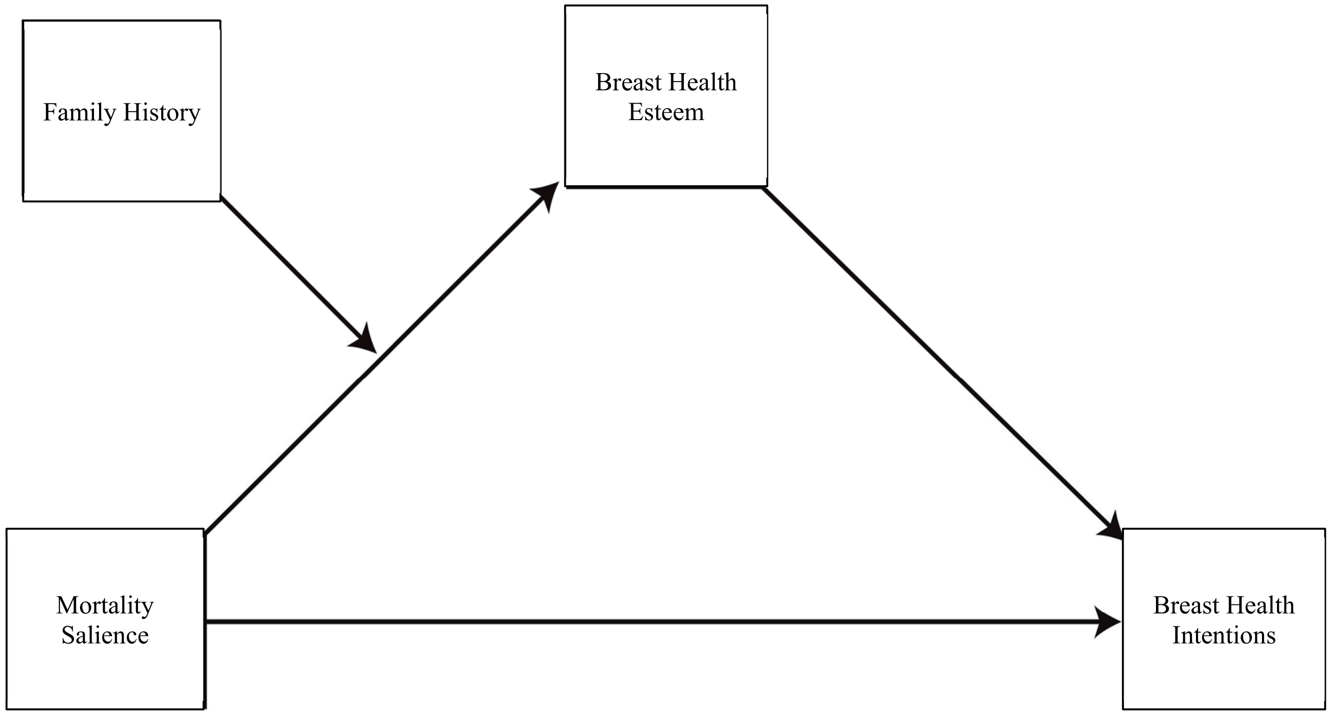

Table S6: Study 2 PROCESS Model 7: Moderated Mediation: MS effects

Controlling for age, race, insurance status, SES, and past BSE behaviors, N = 447

Estimates of effects for serial mediation model.

Model 7:	effect	SE	LLCI	ULCI
Direct Effects on Breast Health Esteem				
MS Condition	−0.140	0.234	−0.600	0.320
Family History				
No family history vs. Survived	0.082	0.263	−0.436	0.599
No family history vs. Died	0.252	0.246	−0.232	0.736
MS x Survived Interaction	0.017	0.357	−0.684	0.719
MS x Died Interaction	−0.308	0.366	−1.026	0.411
Direct Effects on Breast Health Intentions				
MS Condition	0.071	0.852	−0.093	0.235
**Esteem**	**0.287**	**0.027**	**0.234**	**0.340**
Conditional Indirect Effects				
MS → Esteem → Intentions				
No family history	−0.040	0.071	−0.185	0.095
No family history: Survived	−0.035	0.073	−0.179	0.110
Family history: Died	−0.128	0.082	−0.289	0.030
Index of Moderated Mediation				
MS → Esteem → Intentions				
No family history vs. Survived	0.005	0.102	−0.193	0.208
No family history vs. Died	−0.088	0.109	−0.294	0.142

Note: 95% confidence intervals (CI) that do not include zero are considered statistically significant and denoted in **bold**. For comparisons of family history, no family history is coded as 1, family history: survived is coded as 2, and family history: died is coded as 3. The no family history group is treated as the reference category in this analysis, so the other groups are compared to that reference category.

Additional Study 2 MS Analyses

Per research from Lambert and colleagues [63], we performed a 2 (MS: death vs. television) × 3 (family history: none, yes/survived, yes/died) on the positive affect, negative affect, and fear (scared, afraid, jittery, nervous) subscales of the PANAS-SF to determine if subsequent effects could be impacted by fear instigated in the MS condition, especially. There were no significant effects on positive or negative affect. Interestingly, there was a significant main effect of MS on fear, $F(1, 447) = 6.28$, $p = 0.013$, $\eta_p^2 = 0.014$. However, given the lack of interaction between MS and family history, as well as the small effect size, the fear subscale and its implications will only be noted further in the online Supplemental Materials Discussion.

In line with the preregistration plan, and in attempts to extend Study 1 and investigate the role of explicit mortality salience, a moderated serial mediation approach using PROCESS Model 83 was implemented to investigate the moderating effect of family history on the impact of MS on breast health intentions through serial mediation with susceptibility perceptions, death association, and breast health esteem. Again, age, race, insurance status, SES, and prior BSE behaviors served as control variables. Five thousand bootstrap samples were used to create a 95% confidence interval (CI) to investigate indirect effects, where effects are considered significant if the CI does not include 0.

In the first iteration of the model, MS was input as the predictor variable (coded as 0 for the television control and 1 for the mortality salience manipulation) death association was input as the first mediator, susceptibility perception as the second mediator, esteem as the third mediator, and family history as the moderator. MS did not have any significant effects across both direct and indirect pathways in the model. Some significant direct effects did emerge aside from MS. First, there was a direct effect of family history on death associations, $B = 1.01$, $SE = 0.28$, 95%CI [0.45, 1.57], where women whose family member died associated breast cancer with death to a greater extent compared to those with no family history. Further, there was a direct effect of death association on susceptibility perceptions, $B = 0.19$, $SE = 0.02$, 95%CI [0.14, 0.24]. There was also a direct effect of death association on breast health esteem, $B = 0.18$, $SE = 0.04$, 95%CI [0.10, 0.26]. Lastly, there was a direct effect of breast health esteem on breast health intentions, $B = 0.29$, $SE = 0.03$, 95%CI [0.23, 0.34]. See Table S7. Generally, associating breast cancer with death to a greater degree (especially among

women whose family member died of breast cancer) served to predict increased perceptions of susceptibility and esteem associated with breast health behaviors. Additionally, the increase in breast health esteem was

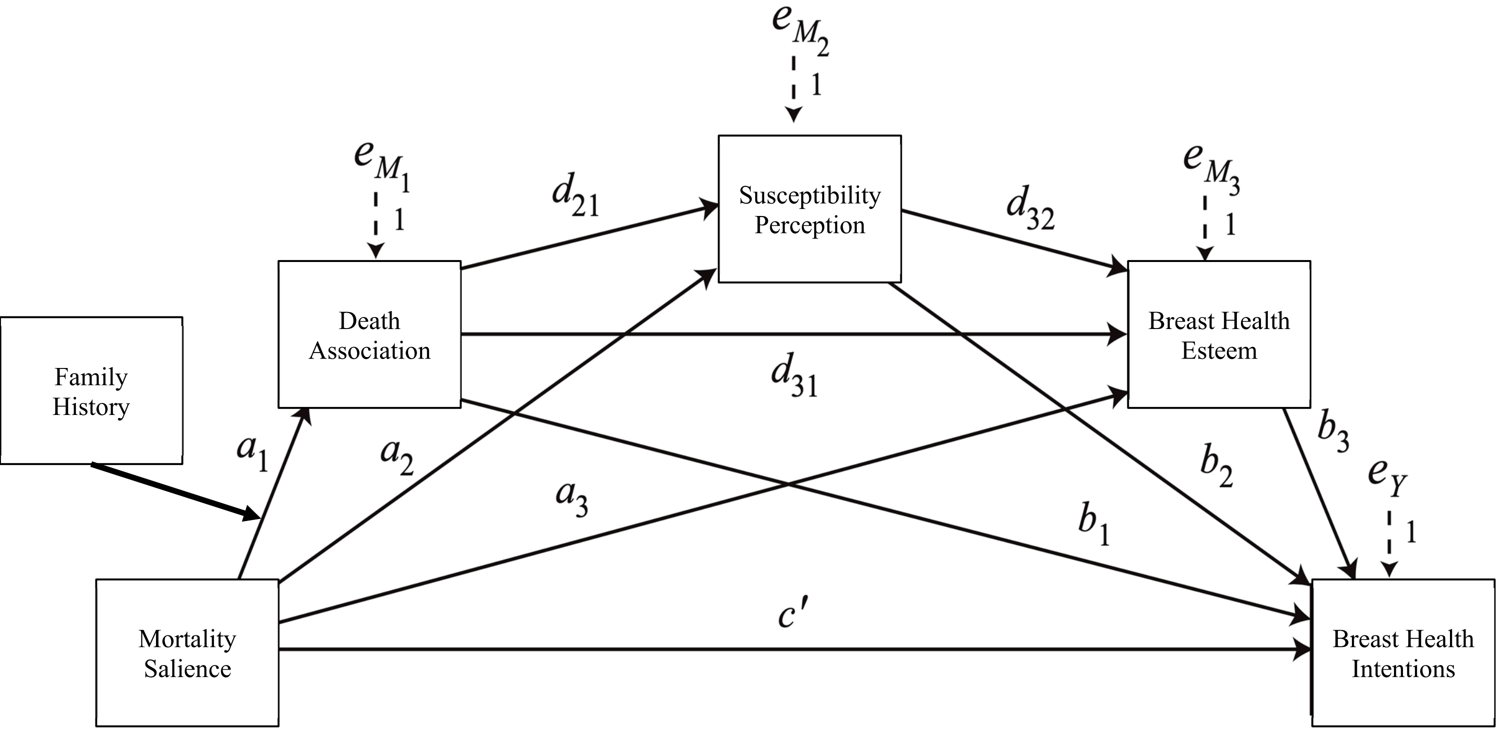

associated with greater breast health intentions. See Figure S3 and Table S5.

Figure S5. *Study 2 PROCESS Model 83 (Death First)*

Table S7. *Study 2 PROCESS Model 83 (Death First)*

Controlling for age, race, insurance status, SES, and past BSE behaviors, N = 447
Estimates of effects for serial mediation model.

Model 83:	effect	SE	LLCI	ULCI
Direct Effects on Death Association				
MS Condition	−152	0.270	−0.379	0.682
Family History				
No family history vs. Survived	0.433	0.304	−0.165	1.030
**No family history vs. Died**	**1.010**	**0.284**	**0.452**	**1.568**
MS x Survived Interaction	−0.546	0.412	−1.355	0.263
MS x Died Interaction	−0.340	0.422	−1.169	0.488
Direct Effects on Susceptibility				
MS Condition	0.037	0.088	−0.135	0.210
**Death Association**	**0.187**	**0.024**	**0.140**	**0.235**
Direct Effects on Breast Health Esteem				
MS Condition	−0.190	0.145	−0.475	0.094
**Death Association**	**0.181**	**0.042**	**0.098**	**0.264**
Susceptibility	0.059	.0079	−0.095	0.214
Direct Effects on Breast Health Intentions				

MS Condition	0.077	0.083	−0.087	0.240
Death Association	0.278	0.025	−0.021	0.076
**Susceptibility**	**−0.110**	**0.045**	**−0.199**	**−0.021**
**Esteem**	**0.287**	**0.027**	**0.234**	**0.341**
Conditional Indirect Effects				
MS → Death Association → Intentions				
No family history	0.004	0.010	−0.017	0.027
Family history: Survived	−0.011	0.016	−0.052	0.013
Family history: Died	−0.005	0.013	−0.037	0.017
Index of Moderated Mediation				
MS → Death Association → Intentions				
No family history vs. Survived	−0.015	0.020	−0.063	0.021
No family history vs. Died	−0.009	0.017	−0.050	0.023
Conditional Indirect Effects				
MS → Susceptibility → Intentions	−0.004	0.010	−0.026	0.016
MS → Esteem → Intentions	−0.055	0.042	−0.140	0.027
Conditional Serial Indirect Effect				
MS → Death Association → Susceptibility → Intentions				
No family history	−0.003	0.006	−0.017	0.009
Family history: Survived	0.008	0.008	−0.004	0.028
Family history: Died	0.004	0.007	−0.009	0.021
Index of Moderated Mediation				
MS → Death Association → Susceptibility → Intentions				
No family history vs. Survived	0.011	0.011	−0.006	0.037
No family history vs. Died	0.007	0.010	−0.010	0.030
Conditional Serial Indirect Effects				
MS → Death Association → Esteem → Intentions				
No family history	0.008	0.015	−0.020	0.041
Family history: Survived	−0.021	0.018	−0.062	0.011
Family history: Died	−0.010	0.018	−0.050	0.021
Index of Moderated Mediation				
MS → Death Association → Esteem → Intentions				
No family history vs. Survived	−0.028	0.025	−0.083	0.015
No family history vs. Died	−0.018	0.024	−0.073	0.022
Serial Indirect Effect				
MS → Susceptibility → Esteem → Intentions	0.000	0.003	−0.004	0.007
Conditional Serial Indirect Effect				
MS → Death Association → Susceptibility → Esteem → Intentions				
No family history	0.001	0.002	−0.003	0.004
Family history: Survived	−0.001	0.003	−0.008	0.003
Family history: Died	−0.001	0.002	−0.005	0.003
Index of Moderated Mediation				
MS → Death Association → Susceptibility → Esteem → Intentions				
No family history vs. Survived	−0.002	0.003	−0.010	0.004
No family history vs. Died	−0.001	0.003	−0.007	0.004

Note: 95% confidence intervals (CI) that do not include zero are considered statistically significant and denoted in **bold**. For comparisons of family history, no family history is coded as 1, family history: survived is coded as 2, and family history: died is coded as 3. The no family history group is treated as the reference category in this analysis, so the other groups are compared to that reference category.

In the second iteration of the model, susceptibility perception was input as the first mediator, death association as the second, esteem as the third, and family history as the moderator, with MS still serving as the predictor variable and intentions as the dependent variable. Again, MS did not have any significant direct nor indirect effects. Some other significant direct effects did emerge. First, there was a direct effect of susceptibility perception on death association, $B = 0.65$, $SE = 0.08$, 95%CI [0.49, 0.82]. There was also a direct effect of death association on breast health esteem, $B = 0.18$, $SE = 0.04$, 95%CI [0.10, 0.26]. There were direct effects of susceptibility perceptions ($B = -0.11$, $SE = 0.05$, 95%CI [−0.20, −0.02]) and esteem on breast health intentions ($B = 0.29$, $SE = 0.03$, 95%CI [0.23, 0.34]). See Figure S6 and Table S8. Interestingly, susceptibility was negatively related to intentions, which could indicate a level of reactance. However, and importantly, increased breast health esteem predicted breast health behaviors.

In these analyses, MS did not have any effects, but the direct effects that did arise lend support to the findings in Study 1, where death associations and susceptibility connected with family history comparisons to facilitate breast health behavioral intentions when those behaviors relate to feelings of esteem.

Figure S6. *Study 2 PROCESS Model 83 (Susceptibility First)*

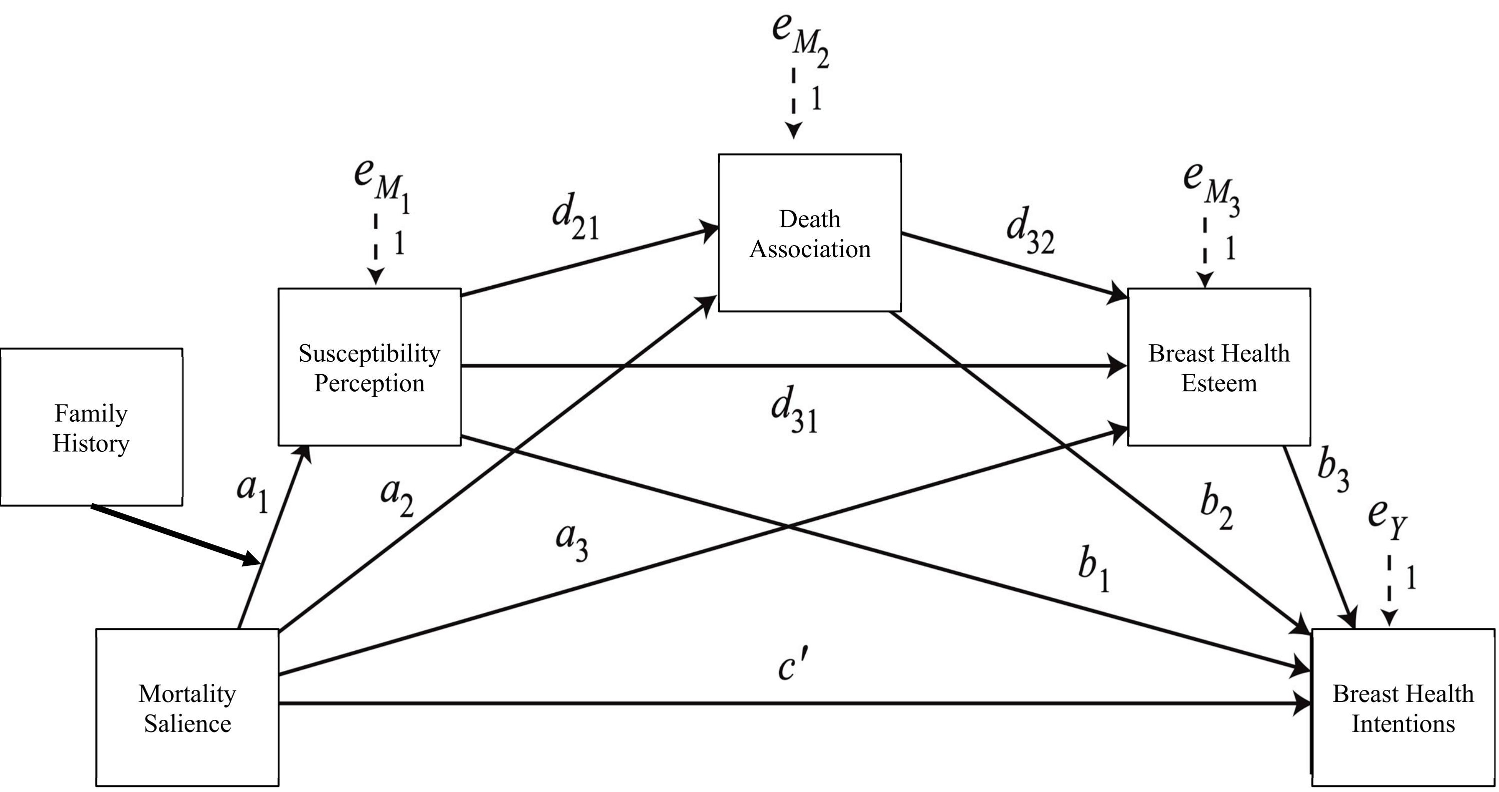

Table S8. *Study 2 PROCESS Model 83 (Susceptibility First)*

Controlling for age, race, insurance status, SES, and past BSE behaviors, N = 447
Estimates of effects for serial mediation model.

Model 83:	effect	SE	LLCI	ULCI
Direct Effects on Susceptibility Perception				
MS Condition	0.148	0.129	−0.125	0.420
Family History				
**No family history vs. Survived**	**0.722**	**0.156**	**0.416**	**1.029**
**No family history vs. Died**	**0.845**	**0.146**	**0.558**	**1.131**
MS x Survived Interaction	−0.304	0.211	−0.719	0.111
MS x Died Interaction	−0.130	0.217	−0.556	0.295
Direct Effects on Death Association				
MS Condition	−0.172	0.164	−0.493	0.149
**Susceptibility Perception**	**0.652**	**0.083**	**0.488**	**0.815**
Direct Effects on Breast Health Esteem				
MS Condition	−0.190	0.145	−0.475	0.094
Susceptibility Perception	0.059	0.079	−0.095	0.214
**Death Association**	**0.181**	**0.042**	**0.098**	**0.264**
Direct Effects on Breast Health Intentions				
MS Condition	0.077	0.083	−0.087	0.240
**Susceptibility Perception**	**−0.110**	**0.045**	**−0.199**	**−0.021**
Death Association	0.028	0.025	−0.210	0.076
**Esteem**	**0.287**	**0.027**	**0.234**	**0.341**
Conditional Indirect Effects				
MS → Susceptibility → Intentions				
No family history	−0.016	0.017	−0.054	0.013
Family history: Survived	0.017	0.020	−0.018	0.065
Family history: Died	−0.002	0.020	−0.045	0.037
Index of Moderated Mediation				
MS → Susceptibility → Intentions				
No family history vs. Survived	0.034	0.028	−0.010	0.098
No family history vs. Died	0.014	0.026	−0.038	0.068

Conditional Indirect Effects				
MS → Death Association → Intentions	−0.005	0.008	−0.025	0.006
MS → Esteem → Intentions	−0.055	0.043	−0.141	0.029
Conditional Serial Indirect Effect				
MS → Susceptibility → Death Association → Intentions				
No family history	0.003	0.004	−0.004	0.013
Family history: Survived	−0.003	0.005	−0.015	0.004
Family history: Died	0.000	0.004	−0.008	0.010
Index of Moderated Mediation				
MS → Susceptibility → Death Association → Intentions				
No family history vs. Survived	−0.006	0.007	−0.024	0.006
No family history vs. Died	−0.002	0.006	−0.015	0.010
Conditional Serial Indirect Effects				
MS → Susceptibility → Esteem → Intentions				
No family history	0.003	0.005	−0.006	0.015
Family history: Survived	−0.003	0.006	−0.016	0.007
Family history: Died	0.000	0.005	−0.010	0.011
Index of Moderated Mediation				
MS → Susceptibility → Esteem → Intentions				
No family history vs. Survived	−0.005	0.009	−0.028	0.010
No family history vs. Died	−0.002	0.007	−0.019	0.101
Serial Indirect Effect				
MS → Death Association → Esteem → Intentions	−0.009	0.009	−0.029	0.008
Conditional Serial Indirect Effect				
MS → Susceptibility → Death Association → Esteem → Intentions				
No family history	0.005	0.005	−0.004	0.016
	effect	SE	LLCI	ULCI
Family history: Survived	−0.005	0.006	−0.020	0.005
Family history: Died	0.001	0.006	−0.012	0.012
Index of Moderated Mediation				
MS → Death Association → Susceptibility → Esteem → Intentions				
No family history vs. Survived	−0.010	0.008	−0.029	0.003
No family history vs. Died	−0.004	0.008	−0.022	0.011

Note: 95% confidence intervals (CI) that do not include zero are considered statistically significant and denoted in **bold**. For comparisons of family history, no family history is coded as 1, family history: survived is coded as 2, and family history: died is coded as 3. The no family history group is treated as the reference category in this analysis, so the other groups are compared to that reference category.

Study 2: Death Association First in Model 6 Replication Analysis

In attempting to replicate findings from Study 1, the same PROCESS Model 6 for serial mediation using death association, susceptibility perception and breast health esteem as mediators on breast health intentions were evaluated with family history used as the sole predictor. In this model, family history was input as the predictor (coded with no family history = 0, family history/survived = 1, and family history/death = 2), death association as the first mediator, susceptibility perceptions as the second mediator, breast health esteem as the third mediator, and breast health intentions as the dependent variable. First, there was a direct effect of family history on death associations among women whose family member died, $B = 0.85$, $SE = 0.21$, 95%CI [0.44, 1.27]. There were also anticipated direct effects on susceptibility perceptions, such that women with any breast cancer

family history, regardless of survival status, perceived themselves as more susceptible (B = 0.54, SE = 0.10, 95%CI [0.34, 0.72] for women whose family survived; B = 0.64, SE = 0.10, 95%CI [0.44, 0.84] for women whose family member died). There was also a direct effect of death association on susceptibility perception, B = 0.16, SE = 0.02, 95%CI [0.12, 0.21]. There was also a direct effect of death association on breast health esteem, B = 0.19, SE = 0.04, 95%CI [0.10, 0.27]. On the breast health intentions variable, there were direct effects of susceptibility perceptions (B = −0.10, SE = 0.05, 95%CI [−0.20, −0.01] and breast health esteem (B = 0.29, SE = 0.03, 95%CI [0.23, .034]). Interestingly, the indirect effect of family history through susceptibility perceptions on intentions was also significant among both family history groups (B = −0.06, SE = 0.03, 95%CI [−0.11, −0.00] for women whose family survived and B = −0.07, SE = 0.03, 95%CI [−0.13, −0.00] for women whose family member died). Further, there was an indirect effect of family history on breast health intentions through death associations and then susceptibility among women whose family member died, B = −0.01, SE = 0.01, 95%CI [−0.03, −0.00]. See Figure S7 and Table S9.

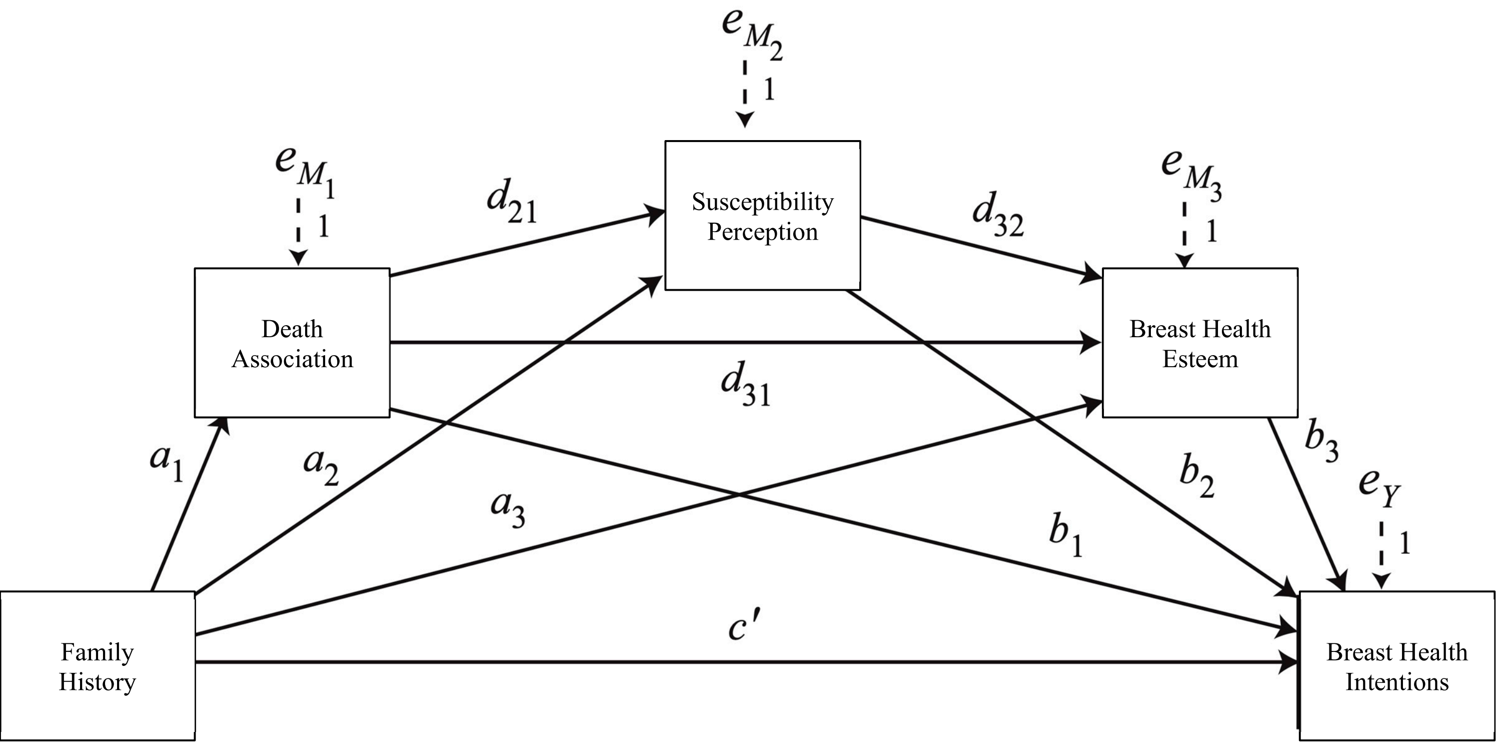

Figure S7. *Study 2 Replication, PROCESS Model 6 (Death First)*

Table S9. *Study 2 Replication, PROCESS Model 6 (Death First)*

Controlling for age, race, insurance status, SES, and past BSE behaviors, N = 447
Estimates of effects for serial mediation model.

Model 6:	effect	SE	LLCI	ULCI
Direct Effects on Death Association				
Family History (a_1)				
No family history vs. Survived	0.128	0.204	−0.272	0.529
**No family history vs. Died**	**0.854**	**0.210**	**0.442**	**1.266**
Direct Effects on Susceptibility Perceptions				
Family History (a_2)				
**No family history vs. Survived**	**0.537**	**0.099**	**0.341**	**0.732**
**No family history vs. Died**	**0.640**	**0.104**	**0.435**	**0.844**
**Death Association (d_{21})**	**0.164**	**0.023**	**0.118**	**0.209**
Direct Effects on Breast Health Esteem				
Family History (a_3)				
No family history vs. Survived	0.022	0.178	−0.328	0.373
No family history vs. Died	−0.079	0.189	−0.450	0.292
Death Association (d_{31})	0.187	0.043	0.103	0.270
Susceptibility Perceptions (d_{32})	0.063	0.083	−0.100	0.226
Direct Effects on Breast Health Intentions				
Family History (c')				
No family history vs. Survived	0.061	0.102	−0.140	0.261
No family history vs. Died	−0.115	0.108	−0.327	0.097
Death Association (b_1)	0.032	0.025	−0.017	0.081
**Susceptibility Perceptions (b_2)**	**−0.103**	**0.048**	**−0.196**	**−0.010**
**Breast Health Esteem (b_3)**	**0.285**	**0.027**	**0.231**	**0.338**
Indirect Effects				
Family history → Death Association → Intentions (a_1b_1)				
No family history vs. Survived	0.004	0.009	−0.011	0.025
No family history vs. Died	0.028	0.024	−0.017	0.078
Family history → Susceptibility → Intentions (a_2b_2)				
No family history vs. Survived	−0.055	0.028	−0.115	−0.003
**No family history vs. Died**	**−0.066**	**0.033**	**−0.133**	**−0.004**
Family history → Esteem → Intentions (a_3b_3)				
No family history vs. Survived	0.006	0.050	−0.091	0.104
No family history vs. Died	−0.023	0.056	−0.131	0.092
Family history → Death → Susceptibility → Intentions ($a_1d_{21}b_2$)				
No family history vs. Survived	−0.002	0.004	−0.011	0.005
**No family history vs. Died**	**−0.014**	**0.008**	**−0.031**	**−0.001**
Family history → Death → Esteem → Intentions ($a_1d_{31}b_3$)				
No family history vs. Survived	0.007	0.012	−0.015	0.032
**No family history vs. Died**	**0.045**	**0.017**	**0.017**	**0.086**
Family history → Susceptibility → Esteem → Intentions ($a_2d_{32}b_3$)				
No family history vs. Survived	0.010	0.013	−0.015	0.036
No family history vs. Died	0.012	0.015	−0.018	0.044
Family history → Death Association → Susceptibility → Esteem → Intentions ($a_1d_{21}d_{32}b_3$)				
No family history vs. Survived	0.000	0.001	−0.002	0.003
No family history vs. Died	0.003	0.003	−0.004	0.010

Note: 95% confidence intervals (CI) that do not include zero are considered statistically significant and denoted in **bold**. For comparisons of family history, no family history is coded as 1, family history: survived is coded as 2, and family history: died is coded as 3. The no family history group is treated as the reference category in this analysis, so the other groups are compared to that reference category.

Supplemental Discussion Point: Lack of MS Effects

Casual, non-systematic evaluations of text responses revealed an interesting thematic pattern. Approximately a third of responses in the death reminder condition included some mention of religion or an afterlife. Select phrases include “my spirit will go to heaven,” “I will be free of this body,” and “the physical body dies, releasing our eternal soul to return back to our Creator.” The notion of religiosity in health behaviors, and through an existential lens, constitutes a double-edged sword. On one hand, attending religious services is a predictor of engagement in cancer screenings (including mammograms and PAP smears), but the relationship between church attendance and screening engagement is contingent on social support(Leyva et al., 2015). In another study, adherence to mammograms and clinical breast exams was associated with lower levels of religiosity among orthodox religious groups (Freund et al., 2019). Further, religious health fatalism (i.e., the belief that death is inevitable and God is in control) is significantly higher among Black/African American people (especially women), and can contribute to poorer management of chronic illnesses (Franklin et al., 2007).

In the context of the present study, it could be the case that mortality salience could have instigated religious responses as a buffering function, which could partially explain the lack of MS effects on breast health esteem, especially. For example, mortality salience can heighten fundamentalist religious beliefs and lead to beliefs that prayer can substitute for medical attention, as well as endorsements for refusals of medical treatment(Vess et al., 2009). Given the lack of MS effects on esteem and intention variables, a reliance on God and the afterlife may have produced a level of religious cancer fatalism in the study sample, which could have given rise to alternate terror-buffering mechanisms. Where death awareness is being buffered by religious beliefs from which esteem can be drawn, it follows that relying on health behaviors for esteem would be secondary or irrelevant. Future TMHM research should better consider religious factors and their influence on health behaviors.

In the same vein as limitation association with the religiosity third variable problem, another limitation is the unclear role of fear, as measured by the PANAS-SF delay/distraction task. Those in the mortality condition reported higher fear-related affect than those in the television-watching control. But, in the critical

preregistered and exploratory mediation and moderated mediation analyses, including fear as a covariate did not contribute to any meaningful differences or changes in significance in the pathways in the models. Despite the lack of significant differences, fear could still be contributing a fair amount of noise to the dependent variables of interest, especially the susceptibility variable and its relationships to breast health esteem and intentions. It makes sense that women who feel susceptible to a deadly health threat like breast cancer might feel a bit more frightened at a state level during a study such as this. Future research in the TMHM could benefit from increasing focus on the role of fear.

Supplemental Tables

Supplemental Table S10: Breast Health Esteem Correlations

Variables: Study 1	1	2	3
1. Taking care of my breast health is an important part of who I am.	-		
2. Taking care of my breast health affects how good I feel about myself.	0.75**	-	
3. Taking care of my breast health allows me to express my competence.	0.70**	0.80**	-
Mean	4.88	4.36	4.23
SD	1.57	1.73	1.77
**Variables: Study 2**	**1**	**2**	**3**
1. Taking care of my breast health is an important part of who I am.	-		
2. Taking care of my breast health affects how good I feel about myself.	0.78**	-	
3. Taking care of my breast health allows me to express my competence.	0.72**	0.82**	-
Mean	4.67	4.25	3.96
SD	1.77	1.76	1.87

Note: * Indicates $p < .05$, ** indicates $p < .001$

Table S11: Breast Health Intentions Correlations

Variables	1	2	3	4	5	6	7	9	10	11	12	13	14	15
Study 1														
In the future														
1. Eat a healthy diet	–													
2. Exercise for 30 minutes 3 times a week	0.69**	–												
3. Go get a mammogram	0.12	0.19**	–											
4. Ask for a clinical breast exam during you well-women's checkup	0.20**	0.26**	0.66**	–										
5. Do a breast self-exam	0.18**	0.07	0.11	0.21**	–									
In the next 3 months														
6. Eat a healthy diet	0.92**	0.67**	0.12	0.24**	0.16*		–							
7. Exercise for 30 minutes 3 times a week	0.65**	0.94**	0.22**	0.28**	0.07	0.70**								
8. Go get a mammogram	0.25**	0.19**	0.46**	0.43**	0.14*	0.24*	0.22**	–						
9. Ask for a clinical breast exam during your well-women's checkup	0.27**	0.22**	0.40**	0.63**	0.26*	0.26**	0.23**	0.70**						
10. Do a breast self-exam	0.20**	0.11	0.16*	0.30**	0.88**	0.22**	0.12	0.22**	0.36**	-				
In the next week														
11. Eat a healthy diet	0.83**	0.67**	0.11	0.18**	0.15*	0.89**	0.67**	0.17*	0.21**	0.20**	-			
12. Exercise for 30 minutes 3 times a week	0.59**	0.88**	0.15*	0.25**	0.08	0.63**	0.89**	0.16*	0.19**	0.14*	0.69**	-		
13. Go get a mammogram	0.16*	0.15*	0.19**	0.24**	0.26**	0.14*	0.16*	0.44**	0.46**	0.27**	0.12*	0.19**	-	

14. Ask for a clinical breast exam during your well-women's checkup	0.19**	0.16**	0.20**	0.26**	0.28**	0.17*	0.20**	0.46**	0.49**	0.30**	0.16*	0.22**	0.92**	-
15. Do a breast self-exam	0.21**	0.13	0.13	0.21**	0.70**	0.21**	0.13	0.27**	0.32**	0.74**	0.22**	0.23**	0.43**	0.46**
Mean	5.61	5.33	5.96	5.53	5.44	5.71	5.38	4.15	4.22	5.20	5.55	4.89	1.91	1.99
SD	1.24	1.63	1.72	1.86	1.78	1.33	1.68	2.45	2.36	1.92	1.56	2.08	1.80	1.88
Variables	1	2	3	4	5	6	7	9	10	11	12	13	14	15
							Study 2							
In the future														
Eat a healthy diet	–													
Exercise for 30 minutes 3 times a week	0.60**	–												
Go get a mammogram	0.15**	0.09**	–											
Ask for a clinical breast exam during you well-women's checkup	0.18**	0.13**	0.66**	–										
Do a breast self-exam	0.25**	0.14**	0.31**	0.41**	–									
In the next 3 months														
Eat a healthy diet	0.89**	0.60**	0.12**	0.14**	0.20**	-								
Exercise for 30 minutes 3 times a week	0.58**	0.94**	0.09	0.11**	0.14**	0.63**								
Go get a mammogram	0.18**	0.09	0.39**	0.36**	0.33*	0.15**	0.10*	–						
Ask for a clinical breast exam during your well-women's checkup	0.23**	0.14**	0.36**	0.55**	0.36**	0.21**	0.14**	0.76**						
Do a breast self-exam	0.25**	0.18**	0.26**	0.41**	0.90**	0.21**	0.18**	0.37**	0.42**	-				
In the next week														
Eat a healthy diet	0.77**	0.56**	0.09	0.15**	0.17**	0.84**	0.57**	0.13**	0.21**	0.20**	-			
Exercise for 30 minutes 3 times a week	0.51**	0.84**	0.04	0.11*	0.13**	0.56**	0.86**	0.08	0.13**	0.19**	0.64**	-		
Go get a mammogram	0.10*	0.02	0.10*	0.14**	0.19**	0.09	0.02	0.38**	0.36**	0.20**	0.09	0.05	-	
Ask for a clinical breast exam during your well-women's checkup	0.11*	0.07	0.10*	0.21**	0.16**	0.11*	0.07	0.32**	0.41**	0.16**	0.15**	0.10*	0.76**	-
Do a breast self-exam	0.14**	0.11*	0.22**	0.30**	0.63**	0.11*	0.13**	0.35**	0.42**	0.69**	0.12**	0.15**	0.36**	0.33**
Mean	5.45	5.23	5.78	5.19	5.33	5.53	5.24	3.91	3.70	5.10	5.20	4.70	1.65	1.72
SD	1.29	1.68	1.79	1.98	1.80	1.36	1.75	2.44	2.27	2.04	1.76	2.11	1.42	1.48

Note: * Indicates $p < .05$, ** indicates $p < .001$

References

American Cancer Society Guidelines for the Early Detection of Cancer. (n.d.). American Cancer Society.

Arndt, J., Cook, A., Goldenberg, J. L., & Cox, C. R. (2007). Cancer and the threat of death: The cognitive dynamics of death-thought suppression and its impact on behavioral health intentions. *Journal of Personality and Social Psychology*, *92*(1), 12–29. https://doi.org/10.1037/0022−3514.92.1.12

Arndt, J., Schimel, J., & Goldenberg, J. L. (2003). Death can be good for your health: Fitness intentions as a proximal and distal defense against mortality salience. *Journal of Applied Social Psychology*, *33*(8), 1726–1746. https://doi.org/10.1111/j.1559−1816.2003.tb01972.x

Brewer, H. R., Jones, M. E., Schoemaker, M. J., Ashworth, A., & Swerdlow, A. J. (2017). Family history and risk of breast cancer: an analysis accounting for family structure. *Breast Cancer Research and Treatment*, *165*(1), 193–200. https://doi.org/10.1007/s10549−017−4325−2

Franklin, M. D., Schlundt, D. G., McClellan, L. H., Kinebrew, T., Sheats, J., Belue, R., Brown, A., Smikes, D., Patel, K., & Hargreaves, M. (2007). Religious fatalism and its association with health behaviors and outcomes. *American Journal of Health Behavior*, *31*(6), 563–572. https://doi.org/10.5993/AJHB.31.6.1

Freund, A., Cohen, M., & Azaiza, F. (2019). Factors associated with routine screening for the early detection of breast cancer in cultural-ethnic and faith-based communities. *Ethnicity & Health*, *24*(5), 527–543. https://doi.org/10.1080/13557858.2017.1346176

Howlader, N., Noone, A. M., Krapcho, M., Miller, D., Brest, A., Yu, M., Ruhl, J., Tatalovich, Z., Mariotto, A., Lewis, D. R., Chen, H. S., Feuer, E. J., & Cronin, K. A. (2019). *SEER Cancer*

Statistics Review, 1975−2017.

Leyva, B., Nguyen, A. B., Allen, J. D., Taplin, S. H., & Moser, R. P. (2015). Is religiosity associated with cancer screening? Results from a national survey. *Journal of Religion and Health*, *54*(3), 998–1013. https://doi.org/10.1007/s10943−014−9843−1

Morris, K. L., Cooper, D. P., Goldenberg, J. L., Arndt, J., & Routledge, C. (2013). Objectification as self-affirmation in the context of a death-relevant health threat. *Self and Identity*, *12*(6), 610–620. https://doi.org/10.1080/15298868.2012.718862

Padamsee, T. J., Muraveva, A., Yee, L. D., Wills, C. E., & Paskett, E. D. (2020). Experiencing the cancer of a loved one influences decision-making for breast cancer prevention. *Journal of Health Psychology*, *25*(8), 1064–1075. https://doi.org/10.1177/1359105317746480

Ramsey, S. D., Yoon, P., Moonesinghe, R., & Khoury, M. J. (2006). Population-based study of the prevalence of family history of cancer: Implications for cancer screening and prevention. *Genetics in Medicine*, *8*(9), 571–575. https://doi.org/10.1097/01.gim.0000237867.34011.12

Vess, M., Arndt, J., Cox, C. R., Routledge, C., & Goldenberg, J. L. (2009). Exploring the existential function of religion: The effect of religious fundamentalism and mortality salience on faith-based medical refusals. *Journal of Personality and Social Psychology*, *97*(2), 334–350. https://doi.org/10.1037/a0015545
